# Tissue-specific impact of stem-loops and quadruplexes on cancer breakpoints formation

**DOI:** 10.1186/s12885-019-5653-x

**Published:** 2019-05-10

**Authors:** Kseniia Cheloshkina, Maria Poptsova

**Affiliations:** Faculty of Computer Science, National Research University Higher School of Economics, 125319 Moscow, 3 Kochnovsky Proezd, Russia

**Keywords:** Stem-loops, Quadruplexes, Non-B motifs, DNA secondary structures, Cancer genomes, Cancer mutations, Breakpoints, Genome rearrangements, Genome instability, Machine learning models, Logistic regression, Random forest

## Abstract

**Background:**

Chromosomal rearrangements are the typical phenomena in cancer genomes causing gene disruptions and fusions, corruption of regulatory elements, damage to chromosome integrity. Among the factors contributing to genomic instability are non-B DNA structures with stem-loops and quadruplexes being the most prevalent. We aimed at investigating the impact of specifically these two classes of non-B DNA structures on cancer breakpoint hotspots using machine learning approach.

**Methods:**

We developed procedure for machine learning model building and evaluation as the considered data are extremely imbalanced and it was required to get a reliable estimate of the prediction power. We built logistic regression models predicting cancer breakpoint hotspots based on the densities of stem-loops and quadruplexes, jointly and separately. We also tested Random Forest models varying different resampling schemes (leave-one-out cross validation, train-test split, 3-fold cross-validation) and class balancing techniques (oversampling, stratification, synthetic minority oversampling).

**Results:**

We performed analysis of 487,425 breakpoints from 2234 samples covering 10 cancer types available from the International Cancer Genome Consortium. We showed that distribution of breakpoint hotspots in different types of cancer are not correlated, confirming the heterogeneous nature of cancer. It appeared that stem-loop-based model best explains the blood, brain, liver, and prostate cancer breakpoint hotspot profiles while quadruplex-based model has higher performance for the bone, breast, ovary, pancreatic, and skin cancer. For the overall cancer profile and uterus cancer the joint model shows the highest performance. For particular datasets the constructed models reach high predictive power using just one predictor, and in the majority of the cases, the model built on both predictors does not increase the model performance.

**Conclusion:**

Despite the heterogeneity in breakpoint hotspots’ distribution across different cancer types, our results demonstrate an association between cancer breakpoint hotspots and stem-loops and quadruplexes. Approximately for half of the cancer types stem-loops are the most influential factors while for the others these are quadruplexes. This fact reflects the differences in regulatory potential of stem-loops and quadruplexes at the tissue-specific level, which yet to be discovered at the genome-wide scale. The performed analysis demonstrates that influence of stem-loops and quadruplexes on breakpoint hotspots formation is tissue-specific.

**Electronic supplementary material:**

The online version of this article (10.1186/s12885-019-5653-x) contains supplementary material, which is available to authorized users.

## Background

The accumulated data on cancer genomes revealed that along with the point mutations, cancer genomes undergo numerous rearrangements including deletions, inversions, tandem duplications and inter and intra-chromosomal translocations [1–3]. The studies on cancer mutagenesis revealed the association between cancer mutations and epigenetic marks and non-B DNA structures [4–7]. Analysis of almost 700,000 somatic copy-number variant breakpoints from around 2800 cancer genomes demonstrated the enrichment of quadruplexes and DNA regions in the hypomethylated state in the vicinity of cancer breakpoints [8]. Epigenetic features, such as chromatin accessibility and histone modifications of a particular type of cancer together with the replication timing explains up to 86% of the variance in single mutation densities for the selected cancer type [9]. Analysis of association between cancer somatic mutations and different non-B DNA structures, including G-quadruplexes (G4), H-DNA, Z-DNA and direct, inverted, mirror and short tandem repeats, revealed two-fold mutation enrichment of the mutation regions by the non-B motifs and demonstrated that machine-learning models built on the densities of the non-B motifs and epigenetic factors either taken separately or jointly are able to predict the densities of somatic mutations [10].

Cancer genome instabilities are associated with double-strand breaks (DSBs) [1], which in turn were shown to be associated with non-B DNA structures and epigenetic features [4, 11]. Machine-learning models using epigenomic and chromatin context reached good accuracy at 1kB resolution in predicting DSBs with chromatin accessibility, activity, and long-range contacts being the best predictors [11].

For stem-loops (or cruciforms) and quadruplexes to form, it is required that DNA were in a single-stranded state that can happen when it is locally unwound. The regions of locally unwound single-stranded DNA can originate during many processes of normal genome functioning such as replication and transcription. The genome-wide potential to form non-B DNA structures was demonstrated by permanganate/S1 nuclease footprinting [12]. Thousands of non-B motifs were found in the regions of unwound DNA pointing to their role in various processes of genome functioning including transcription and regulation of nucleosome positioning. At the same time the locally unwound regions with emerging non-B DNA structures could cause genome instability.

Here we explored the data on all types of cancer genome rearrangements available from the International Cancer Genome Consortium for the breakpoint association with two most prevalent types of non-B DNA structures – stem-loops and quadruplexes, and studied how this association is varied depending on the type of cancer. We investigated breakpoint chromosome distribution at 6 different resolutions from 10kB to 1 Mb and selected breakpoint hotspots at 5 aggregation levels considering breakpoint hotspots to be the regions with frequent and recurrent rearrangements. We confined our study to two classes of the most prevalent non-B DNA structures – stem-loops (or cruciforms) and quadruplexes since they have the highest coverage in the genome and the highest potential to form in regions of a single-stranded DNA. Since mutational landscapes of cancerous genomes are highly heterogeneous, we investigated each type of cancer separately and also built the generalized cancer genome profile. Our study revealed that for approximately half of the cancer types stem-loops have the larger impact on breakpoint hotspots’ prediction while for the other half the most important contributors are quadruplexes. The different impact of stem-loops and quadruplexes on breakpoint formation in different types of cancer is most likely related to the different impact of these two types of non-B DNA structures in tissue-specific regulation.

## Results

### Breakpoint hotspots

Data on cancer breakpoints were downloaded from the International Cancer Genome Consortium (ICGC) Data Portal (release 25) (see Methods). The available data comprised 10 cancer types containing 2234 samples. After filtering for inaccuracy in breakpoint positions (see Methods) we ended up with 487,425 breakpoints. The number of samples and corresponding number of breakpoints by cancer type are given in Additional file 1: Table S1. The distribution of samples among different cancer types is not uniform. Breast cancer comprises the major part of the dataset (644 samples) while the brain and uterus cancers are represented by a relatively small number of samples (72 and 16 accordingly). The distribution of the number of breakpoints by different types – deletions, insertions, inversions, inter- and intrachromosomal translocations, and others, is presented in Additional file 1: Figure S1 with deletions being on the first place, inversions on the second and intrachromosomal rearrangements with non-inverted orientation on the third.

**Fig. 1:**
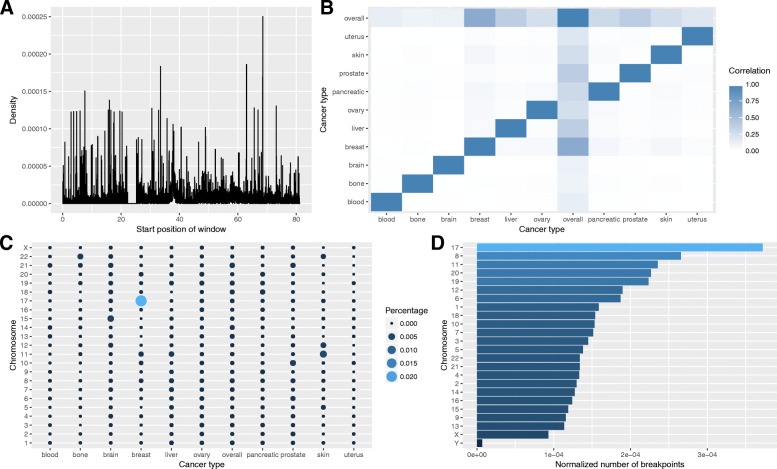
**a** Number of breakpoint hotspots by chromosome and type of cancer for 0.1% labeling type. **b** Correlation between different cancer profiles. **c** Breakpoint density in general cancer profile across chromosome 17 (in Mb). **d** Number of breakpoints normalized by chromosome length

To analize breakpoint distributions across different chromosomes, the number of breakpoints in each chromosome was divided by the length of the chromosome. This normalization allows comparing the breakpoint coverage between different chromosomes. It was revealed (Fig. 1d) that the chromosome 17 has the highest normalized coverage with the chromosome Y being on the last place. Considering a relatively small number of breakpoints it was decided to exclude Y-chromosome from the analysis.

Breakpoint density was calculated for each cancer type (see Methods) for six different windows of 10, 20, 50, 100, 500 Kb and 1 Mb (designated further as aggregation levels). The general breakpoint density profile, or overall cancer profile, accumulating information about all cancer types (see Methods) is presented in Fig. 1c for the chromosome 17. Spearman correlation analysis of breakpoint density profiles for all types of cancers and the overall cancer profile revealed that while particular cancer profiles by definition correlate with the general, no correlation is found in between various types of cancer (Fig. 1b).

We defined breakpoint hotspots according to five different probability thresholds (designated further as labeling types; see Methods) and present results conducted for all five labeling types. In general, the window is marked as a breakpoint hotspot if breakpoints density in the window is higher than the threshold. Thus, for each cancer type and aggregation level we created five datasets with different types of labeling. The distributions of the number of breakpoint hotspots per chromosome for all cancer types and for different thresholds are given in Fig. 1a and Additional file 1: Figure S2. At the 0.1% labeling type, or threshold, the breast cancer has the biggest number of breakpoint hotspots on the chromosome 17, the skin cancer has a relatively high number of hotspots on the chromosomes 11 and 12, the brain cancer – on the chromosome 15, and the bone cancer – on the chromosome 22. The other cancer types have uniform cancer breakpoint hotspots distributions per chromosomes.

We compared breakpoint hotspots’ profiles between different types of cancer using Jaccard similarity coefficient (Additional file 1: Figure S3). This metric shows the ratio of two samples intersection size to their union size and hence demonstrates relative overlap of two samples. Similar to the breakpoint density profiles, hotspots of different cancer types do not intersect with each other; even the general cancer profile does not show high similarity to other cancer types with the largest value of 0.323 for the blood cancer.

**Fig. 2:**
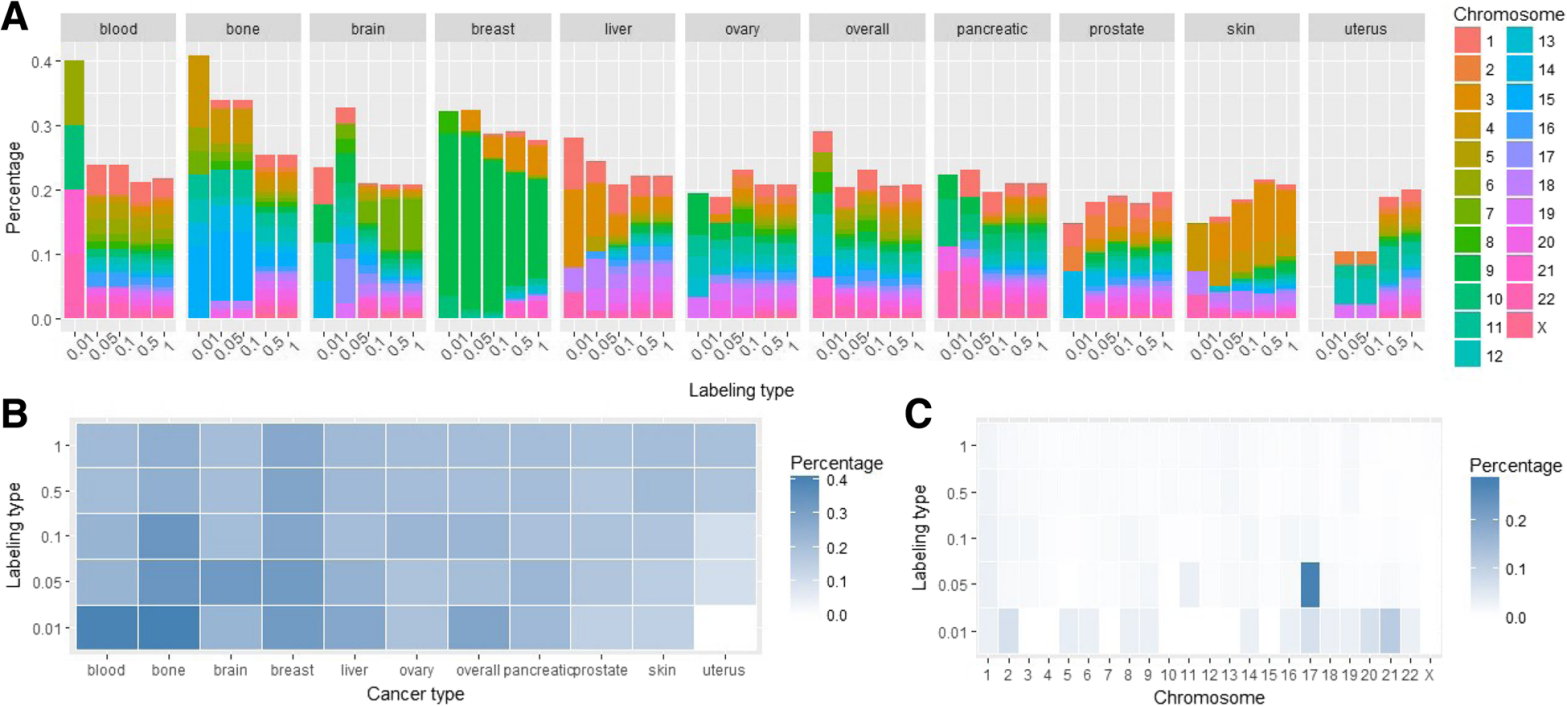
**a** Percentage of breakpoint hotspots intersecting with whole gene regions in different cancer types for 5 different hotspot labeling types. **b** Heatmap of the percentage of the breakpoint hotspots’ intersections with whole gene regions across different cancer types. **c** Heatmap of the percentages of all breakpoint hotspots’ intersections in all cancer types stratified by different chromosomes

**Fig. 3:**
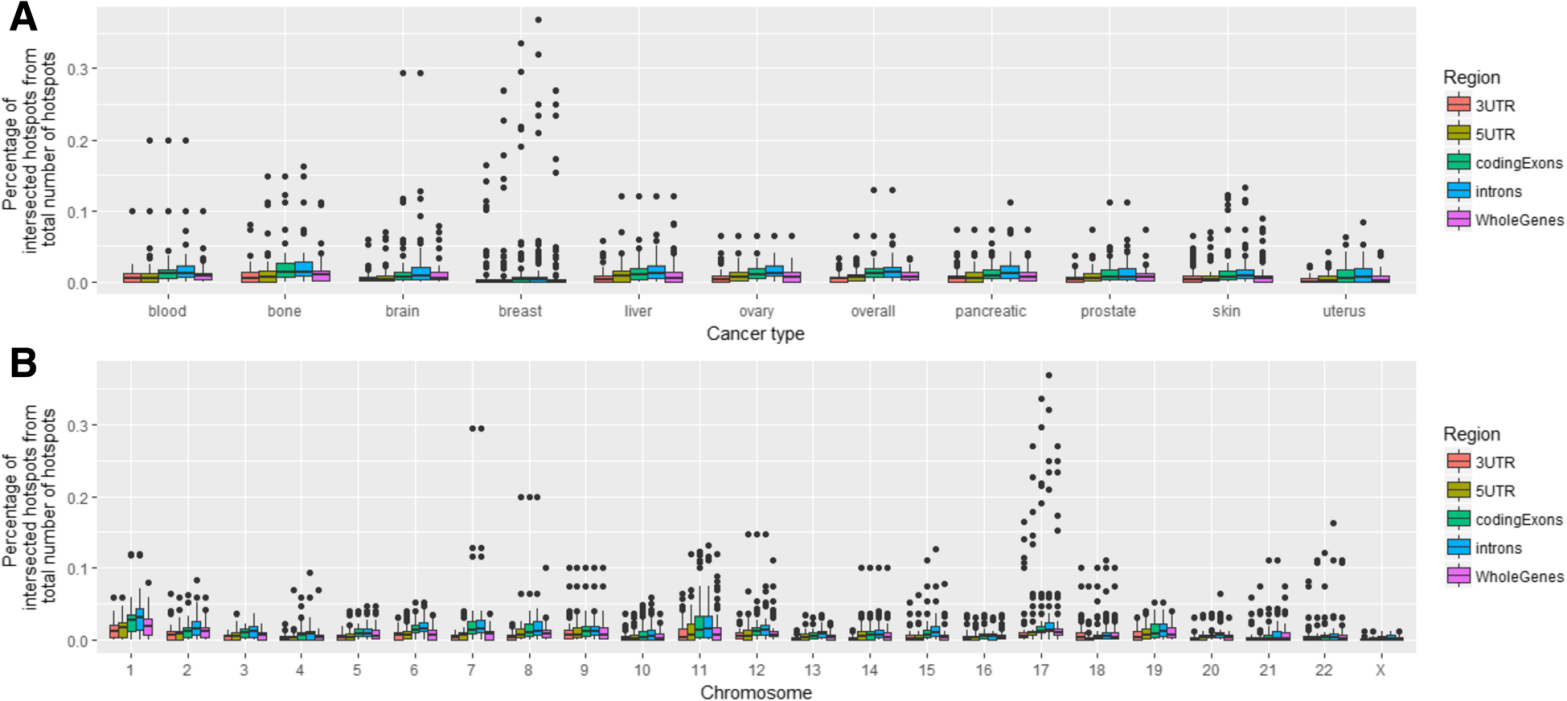
Percentage of breakpoint hotspots intersecting with different genomic regions - whole genes, promoters, downstream regions, and the regions inside genes - 5’UTR, 3’UTR, coding exons and introns in different cancer types (**a**) and in different chromosomes (**b**)

We also checked the distribution of breakpoints and hotspots among different genomic regions, including whole genes, promoters, downstream regions, and the regions inside genes: 5′ untranslated region (5′ UTR), 3′ untranslated region (3′ UTR), coding exons and introns. Almost half of all breakpoints (48%) fall inside genes, though almost all are located inside introns (46%); 1.5% of breakpoints fall into coding regions, and less than 1 % fall into promoters, downstream regions or 5’UTR; 1.7% of all breakpoints fall into 3’UTR (Additional file 1: Figure S4). We checked the intersection of hotspots with whole genes and found that the percentage of breakpoint hotspots overlapping with genes varies from 10% in the uterus cancer to 40% in the blood and bone cancers (Fig. 2a). Overall the highest percentages of hotspots’ overlap with whole genes are observed for the blood, bone, brain, and breast cancer (Fig. 2b). When looking at the distributions of all hotspots from all cancers stratified by the chromosomes, the chromosome 17 shows the maximum overlap (up to 30%) with the genes. The percentage of intersections of breakpoint hotspots with promoters, genes and downstream regions in different types of cancers and in different chromosomes is depicted in Fig. 3. For genomic regions the highest percentage is observed for the breast, brain, blood, and bone cancers, and for chromosomes the highest percentage is observed for the chromosome 17 followed by the chromosomes 7 and 8.

### Correlations with stem-loops and quadruplexes

Here we consider two types of DNA secondary structures - stem-loops and quadruplexes. We distinguish three types of stem-loops according to the size of the stem: short, medium and long, and consider each type as a variable. For each of these structures we calculated coverage as a measure of density (see Methods). Example of stem-loops, quadruplexes and breakpoints distribution for some types of cancer is given in Additional file 1: Figure S5. Spearman correlation between densities of different cancers and stem-loop/quadruplex coverage was calculated separately for each chromosome for 10 kb and 1 Mb aggregation levels (Additional file 1: Figures S6–7).

As for the stem-loops, correlation is higher in general for 1 Mb aggregation level (Additional file 1: Figure S6) with the median correlation increased from 0 (for 10 kb windows) to 0.3 for many types of cancers such as the skin, prostate, uterus, pancreatic, and breast. In addition, for almost all cancer profiles (except for the uterus cancer) there are chromosomes with the correlation higher than 0.6. Correlation distributions among stem-loop classes (short, medium and long stem-loops) do not differ much for different types of cancer.

Similar to stem-loops, correlations between breakpoint densities and quadruplex coverage are higher for 1 Mb aggregation level (Additional file 1: Figure S7). Also it could be noted that there are cancer types with chromosomes that have a low correlation (blood, brain, liver, uterus). The highest median correlation is found for the breast and ovary cancer.

### Machine-learning model building

For all cancer types we explored 6 aggregation levels of 10, 20, 50, 100, 500 kb and 1 Mb and 5 different labeling types with probability thresholds of 0.01, 0.05, 0.1, 0.5, and 1% to select breakpoint hotspots. This led to creation of 330 cancer profiles; the number of breakpoints hotspots by the cancer type, aggregation level and labeling type are given in Additional file 1: Table S2. For some labeling types, the number of breakpoint hotspots at the majority of aggregation levels is not sufficient to build machine learning models, thus these profiles were excluded together with the duplicated profiles, reducing the total number of datasets to 236 (see Methods).

The final datasets were composed from cancer profiles and stem-loop and quadruplex coverage profiles. We built three types of models to predict breakpoint hotspots: based on the stem-loop coverage, on quadruplex coverage, and jointly on stem-loop and quadruplex coverage.

As classes in the considered datasets are extremely imbalanced due to the selected labeling types, a reliable procedure for the prediction power estimation was required. Initially we built Random Forest models varying different resampling schemes (leave-one-out cross validation (LOOCV), train-test split, 3-fold cross-validation) and class balancing techniques (oversampling, stratification, synthetic minority oversampling technique (SMOTE)) (see Methods). We found that the best performance is achieved through the use of 3-fold cross-validation with oversampling. At the same time, we observed overfitting as the relative difference between median area under the receiver operating characteristic curve (ROC AUC) for the train and test sets reached 33%. In order to avoid overfitting as well as inability of class separation we performed 15-times repeated 3-fold cross-validation based on the logistic regression with oversampling (see Methods).

### Stem-loop based models

We built the logistic regression model (see Methods) based on two types of stem-loops – short and long – for all 236 datasets. We excluded medium stem-loops as they have 94% correlation with short stem-loops. Concerning ROC AUC, we calculated two types of confidence intervals for the mean test AUC based on the standard deviation and on the standard error (see Methods). Both confidence intervals agreed in all cases with the only one dataset having the value of 0.5 (Table 1).

**Table 1:** Stem-loop-based ML models

	Stem-loop-based models			
Cancer type	Median test AUC	Percentage of datasets with the mean test AUC confidence interval not containing 0.5	Median lift of recall	Percentage of datasets with the lift of recall higher than 1.5
skin	0.54	100	1.12	22
overall	0.55	100	1.57	52
prostate	0.55	100	1.22	13
uterus	0.55	100	1.22	32
bone	0.56	100	1.00	35
brain	0.59	100	2.07	80
breast	0.57	96	1.57	52
ovary	0.54	100	1.18	14
pancreatic	0.54	100	1.06	5
blood	0.58	100	1.62	60
liver	0.57	100	1.60	57

Performance metrics by cancer type

**Fig. 4:**
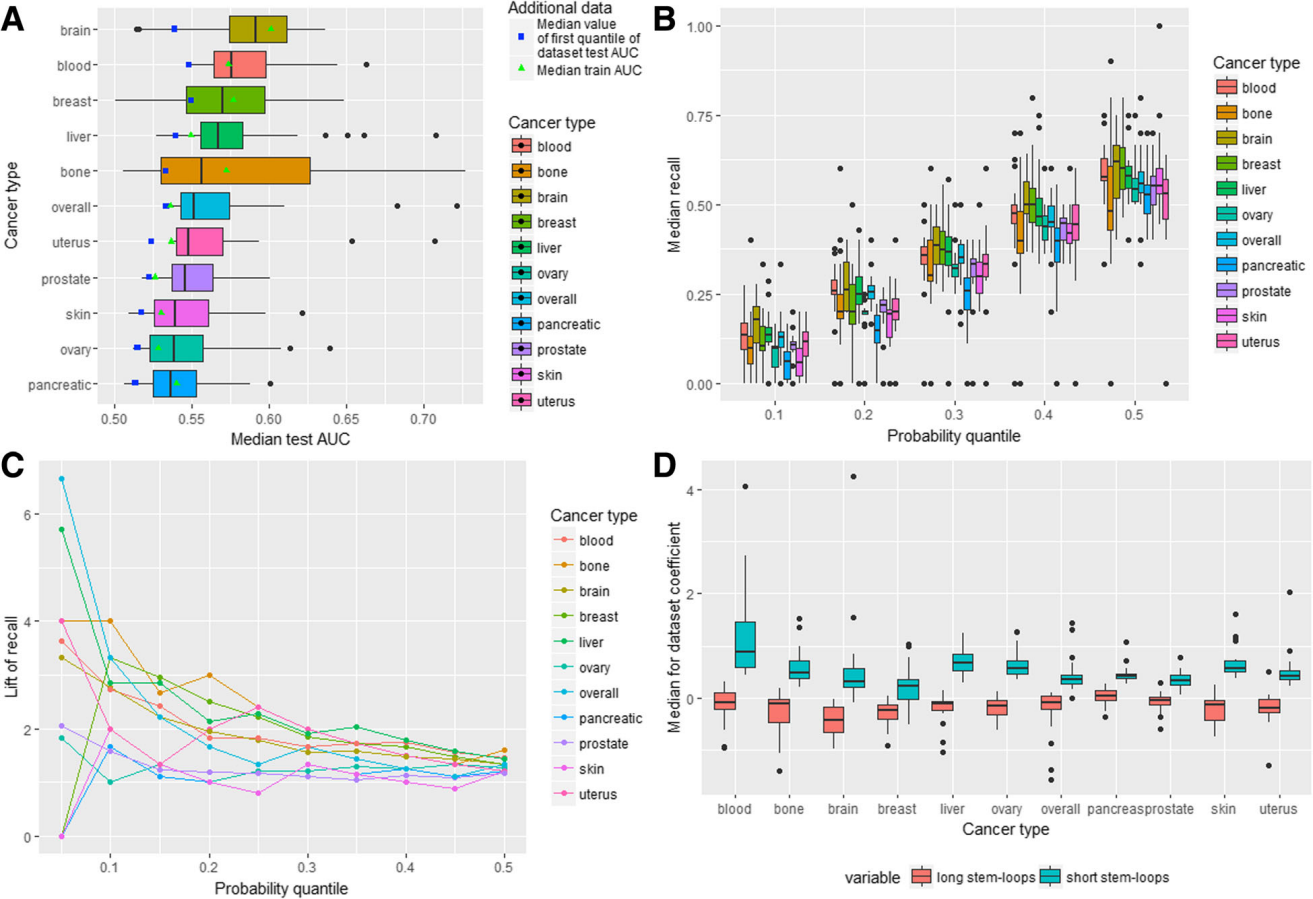
Stem-loop-based ML models. **a** AUC-related metrics for all cancer types. **b** Distribution of dataset median recall for all cancer types. **c** Lift of recall for best models for all cancer types for all probability quantiles. **d** Variable importance for all cancer types

Distributions of the datasets’ median test AUC metric for each cancer type is given in Fig. 4a. There is no cancer type for which all settings (the aggregation levels and labeling types) are equally good or bad. There are cancers, which have outliers with the median test AUC for one particular setting being significantly higher than for the others (blood, ovary, pancreatic, liver, skin, uterus cancers, overall cancer profile). The median test AUC (Table 1) is the highest for the brain cancer and the lowest for the pancreatic cancer. The maximum values of the test AUC > 0.7 are observed for the bone, liver, and uterus cancer. Also it could be noted that the standard deviation of the test AUC is the smallest for the pancreatic, prostate and skin cancers (0.024, 0.027, 0.030 respectively) and it is the highest for the bone cancer (0.069) (Additional file 2). Thus, according to the median test AUC values, the performance of models built on different datasets varies for each cancer type.

We calculated the median value of the standard deviation of the test AUC for each dataset of various cancer types separately and found that it has the minimum value for the skin and pancreatic cancer (0.032 and 0.034 respectively) and the maximum for the brain cancer (0.063). Thus, in general, the models for cancer types with the small number of the analyzed samples are less stable than the others although the liver cancer, which has relatively many samples (255), demonstrates the relatively big median standard deviation (0.047). The analysis of combinations of the labeling type and aggregation level revealed that the median values of the standard deviation of the test AUC and the median test AUC changes in the same direction: the higher the median test AUC the higher the median of the test AUC standard deviation (Additional file 1: Figure S8A).

To keep the balance between the standard deviation and the median of the test AUC the following combinations of the aggregation level and labeling type could be selected: (20 kb, 0.01%); (50 kb, 0.1%); (100 kb, 0.05%); (500 kb, 1%); (1 Mb, 1%). In other words, it is preferable to classify breakpoint hotspots either based on the higher probability threshold with the larger window sizes or on the lower probability threshold with the smaller window sizes.

Distributions of the median recall for all datasets grouped by the different probability quantiles (the probability thresholds at which the given percentage of observations with the maximum probability is selected) and cancer types are given in Fig. 4b. It could be seen that the distributions are very broad so that in all cancer types different settings (the aggregation level and labeling type) result in very diverse recall. For example, considering the probability threshold of 0.2 it could be seen that the median recall of almost all types of cancer including blood, bone, breast, liver, ovary, pancreatic, prostate, skin and uterus cancer ranges from 0 to more than 0.2. It demonstrates that the aggregation level and labeling type considerably impact performance of the model. Additional file 1: Table S3 contains the median and the third quantile of recall (in brackets) for all datasets grouped by cancer type and the probability quantile. It could be seen that in almost all cases the median recall is not much higher than the probability quantile. For the pancreatic and bone cancer the median recall for all probability quantiles is less or equal to a random choice recall.

The lift of recall can provide an estimate of how the model behaves in comparison to a random model and it measures how much the performance of the model is higher in comparison to a random choice (see Methods). Filtering datasets with the lift of recall higher than 1.5, revealed 89 cases (Table 1): 16 for the brain cancer (80% of all brain cancer datasets), 12 for the blood, breast, liver cancer and overall cancer profile (60, 52.18, 57.14, 52.17% respectively), 7 for the bone cancer (35%), 6 for the uterus cancer (31.58%), 5 for the skin cancer (21.74%), 3 for the prostate and ovary cancers (13.04 and 13.64%) and 1 for the pancreatic cancer (4.55%). Among these 89 datasets 17 datasets have 1 Mb aggregation level; 16–100 kb aggregation level; 15–50 kb and 10 kb; 13–20 kb and 500 kb. As for the labeling type, 22 datasets have 0.5% labeling, 21–0.1% labeling, 18–0.05% labeling, 17–1% labeling, 11–0.01% labeling. The best combinations of the labeling type and aggregation level are (1 Mb, 1%) and (1 Mb, 0.5%) (8 and 9 datasets).

After selection of the best threshold for each dataset according to the lift of recall and then calculation of the median lift of recall for each cancer type it was revealed that the maximum median lift of recall is observed for the brain cancer (2.07) and minimal for the bone (1.00) and pancreatic cancer (1.06) (Table 1). Concerning the other cancer types, the overall cancer profile, blood, breast and liver cancer have the median lift of recall higher than 1.5 (1.57, 1.62, 1.57 and 1.60 respectively) while the median lift of recall for the uterus, skin, ovary and prostate cancer under 1.5.

The lift of recall is considered to be a good measure of prediction power. The best models (or datasets, for which the created model achieved the best performance) were selected for each cancer type based on the maximum lift of recall calculated for the given probability quantile. With this approach 11 models were chosen and for each of them the optimal probability quantile was fixed (maximum among all probability quantiles for the model) (Additional file 1: Table S4).

Figure 4c depicts the lift of recall for the best models of all cancer types for all probability quantiles. It could be seen that for all cancer types the highest lift of recall is for the probability quantiles 0.05 and 0.10. For the majority of the cancer types (not including the pancreatic, breast, ovary and skin cancer) the lift of recall is higher than random (higher than 1) for all 10 considered thresholds with 9 such thresholds for the breast cancer profile, 8 – for the ovary cancer, 7 – for the pancreatic cancer and 5 – for the skin cancer. Thus models for some types of cancer perform better than a random choice for all or almost all thresholds (brain, bone, liver, uterus, blood and prostate cancer, overall cancer profile). The lift of recall for the selected 11 models ranges from 1.67 to 6.67 being maximal for the overall cancer profile (6.67), liver (5.71), bone and uterus (4) cancer and minimal for the pancreatic (1.67) and ovary cancer (1.82).

The variable importance analysis based on the logistic regression coefficients for the predictors is depicted in Fig. 4d. In general, the direction of the effect of each type of stem-loops is similar for almost all cancer types. For all cancer types the short stem-loops incorporate the major part of the positive effect with the median coefficient value ranging from 0.23 for the breast cancer to 0.88 for the blood cancer. Concerning long stem-loops, for all except the pancreatic cancer, the median effect is negative being the strongest for the brain cancer (− 0.43) and the weakest for the prostate cancer (− 0.039).

### Quadruplex-based models

**Fig. 5:**
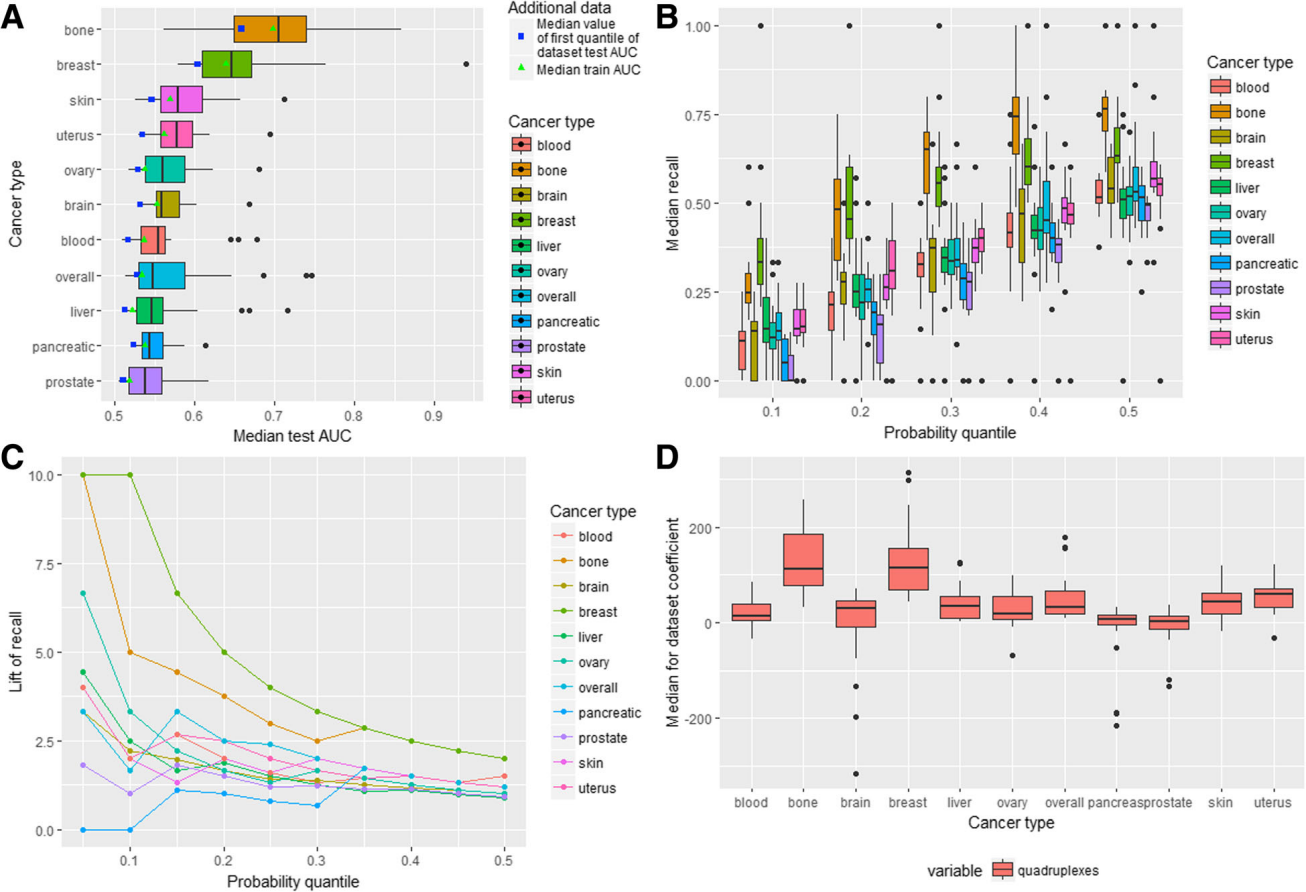
Quadruplex-based ML models. **a** AUC-related metrics for all cancer types. **b** Distribution of datasets median recall for all cancer types. **c** Lift of recall for best models for all cancer types for all probability quantiles. **d** Variable importance for all cancer types

To estimate the prediction power of quadruplexes we built logistic regression models similar to stem-loop models to predict breakpoint hotspots by quadruplex coverage for all 236 datasets. It was revealed that the confidence intervals for the mean test ROC AUC do not include 0.5 for all datasets (Table 2). Distribution of the median test AUC by cancer type is demonstrated in Fig. 5a and Table 2.

**Table 2:** Quadruplex-based ML models

	Quadruplex-based models			
Cancer type	Median test AUC	Percentage of datasets with the mean test AUC confidence interval not containing 0.5	Median lift of recall	Percentage of datasets with lift of recall higher than 1.5
skin	0.58	100	1.69	70
overall	0.55	100	1.58	61
prostate	0.54	100	1.00	9
uterus	0.58	100	1.76	79
bone	0.70	100	3.17	100
brain	0.56	100	1.63	65
breast	0.65	100	3.64	100
ovary	0.56	100	1.31	45
pancreatic	0.54	100	1.21	9
blood	0.55	100	1.32	40
liver	0.55	100	1.13	57

Performance metrics by cancer type

It could be seen that the median test AUC is maximum for the bone and the breast cancer while it is minimal for the prostate cancer (Table 2). At the same time the breast and bone cancer have the biggest standard deviation of the test AUC (0.08 and 0.078 respectively), which is more than 3 times higher than the minimal standard deviation (for pancreatic cancer – 0.023). In general, weak performance is associated with the small standard deviation (a stable weak prediction power) while for cancer types with a relatively high performance dataset configuration is important. Similarly, the median value of the standard deviation in one dataset is the smallest for overall cancer profile and prostate cancer (0.039 and 0.041) and the biggest for the breast and uterus cancer (0.06 and 0.068) taking into account the small uterus cancer sample size (16 samples from 7 donors). The distributions of the median test AUC and the median of the standard deviation in a dataset are presented in Additional file 1: Figure S8B. The optimal combinations of the aggregation level and labeling type are the same as in the case of stem-loop-based models.

Distributions of the median recall for different cancer types and probability thresholds are presented in Fig. 5b. It could be seen that the distributions are broad with outliers at values of 0 and 1. The highest median recall is observed for the bone and breast cancer with the prostate cancer having the lowest median recall for all probability thresholds.

The median recall and third quantile of the distributions are given in Additional file 1: Table S5. For several cancer types (prostate, pancreatic, blood and ovary) the median recall is less or slightly higher than a random choice recall for all probability thresholds. There are 135 datasets (Table 2) with the lift of recall higher than 1.5 including 23 datasets for the breast cancer (100%), 20 datasets for the bone cancer (100%), 16 - for the skin cancer (70%), 15 – for the uterus cancer (79%), 14 – for the overall cancer profile (61%), 13 – for the brain cancer (65%), 12 – for the liver cancer (57%), 10 – for the ovary cancer (45%), 8 – for the blood cancer (40%), 2 – for the prostate and pancreatic cancer (9%).

Selection of the optimal threshold for each dataset (Table 2) leads to the conclusion that the median lift of recall is the highest for the breast and bone cancer (3.64 and 3.17 respectively) and the lowest for prostate and liver cancer (1.00 and 1.21). As for the other types of cancer the median lift of recall is greater than 1.5 for the skin (1.7), uterus (1.76), brain (1.63) cancers and overall cancer profile (1.58).

Among the best models (Fig. 5c and Additional file 1: Table S6), the highest lift of recall is observed for the breast and bone cancer (10 for both) with the test ROC AUC 0.94 and 0.85 respectively. For all cancer types except for the pancreatic and prostate cancers (1.71 and 1.82) the lift of recall ranges from 3.33 to 6.67.

Analysis of logistic regression coefficients’ distribution (Fig. 5d) revealed that in general probabilities of cancer breakpoint hotspots are increasing with the growth in the quadruplex coverage for all cancer types. The largest positive effect is observed for the breast and bone cancer with the median values of the coefficients equal to 115 and 112 respectively. The prostate and pancreatic cancers have the lowest coefficients (1.2 and 6.7). The standard deviation of the coefficients behaves similarly: it is relatively big for the breast and bone cancer and small for the prostate and pancreatic cancer. In addition, for almost all cancer types, except for the bone, breast, liver and overall cancer, there are datasets with negative coefficient estimates.

### Joint stem-loop and quadruplex-based models

**Fig. 6:**
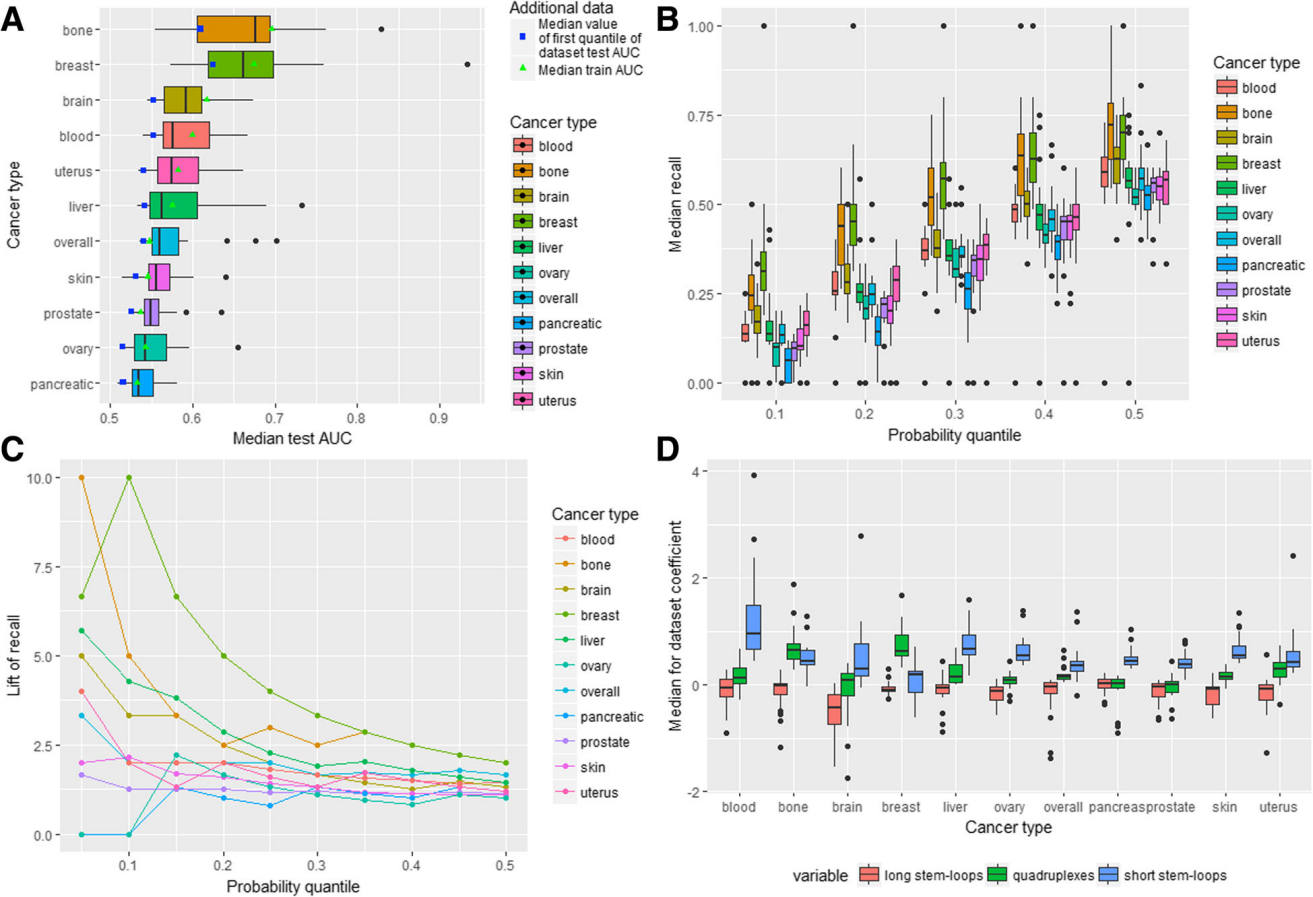
Stem-loops and quadruplex-based ML models. **a** AUC-related metrics for all cancer types. **b** Distribution of datasets median recall for all cancer types. **c** Lift of recall for best models for all cancer types for all probability quantiles. **d**. Variable importance for all cancer types

Finally, in order to get deeper understanding of the relationship between stem-loops and quadruplexes and cancer breakpoints we built models taking into account both stem-loop and quadruplex genome-wide coverage. As in the case of quadruplex-based models, confidence intervals for the mean test AUC do not include 0.5 for all considered datasets (Table 3). Figure 6a demonstrates the median test ROC AUC distribution by cancer type. The bone and breast cancer show distinctive performance following by the brain cancer, and the lowest value is observed for the pancreatic cancer. As in the previous models, the standard deviation of the median test AUC increases with the growth in performance. The median standard deviation in one dataset does not behave likewise and the maximum value is observed for the brain cancer (0.061) while the pancreatic cancer is again described by the minimum value (0.033). Additional file 1: Figure S8C demonstrates that in general specific combinations of the aggregation levels and labeling types for the joint stem-loop and quadruplex-based models are characterized by nearly the same relationship between the median test AUC and the median standard deviation as in the separate models.

**Table 3:** Joint stem-loop and quadruplex-based ML models

	Stem-loops and quadruplex-based models			
Cancer type	Median test AUC	Percentage of datasets with the mean test AUC confidence interval not containing 0.5	Median lift of recall	Percentage of datasets with lift of recall higher than 1.5
skin	0.56	100	1.37	39
overall	0.56	100	1.67	57
prostate	0.55	100	1.21	4
uterus	0.57	100	2.00	68
bone	0.68	100	3.17	100
brain	0.59	100	1.93	90
breast	0.66	100	3.64	100
ovary	0.54	100	1.15	27
pancreatic	0.53	100	1.06	0
blood	0.58	100	1.33	60
liver	0.56	100	1.32	52

Performance metrics by the cancer type

Considering recall, Fig. 6b and Additional file 1: Table S7 show that the pancreatic, ovary, prostate and skin cancer have less or insignificantly higher than random median recall for all probability quantiles taking into account coverage of both stem-loops and quadruplexes. On the other hand, the breast and bone cancer have considerably higher performance than the other cancer types.

As for the lift of recall, there are 126 datasets with the lift of recall higher than 1.5 namely 23 datasets for the breast cancer (100%), 20 – for the bone cancer (100%), 18 – for the brain cancer (90%), 13 – for the uterus cancer and overall cancer profile (68 and 57%), 12 – for the blood (60%), 11 – for the liver (52%), 9 – for the skin (39%), 6 – for the ovary (27%), 1 – for the prostate (4%) and none for the pancreatic cancer (Table 3). Filtering probability thresholds with the maximum lift of recall for each dataset, we ended up with the next values of the median lift of recall: for half of the cancer types (overall cancer profile, uterus, brain, bone, breast) it is higher than 1.5 with the maximum for the breast cancer (3.64). For the pancreatic and ovary cancer it is near 1 (1.06 and 1.15).

Selection of the best models (Additional file 1: Table S8) for each cancer type according to the maximum lift of recall leads to the following results: for half of the cancer types the lift of recall is not less than 4 (uterus, bone, brain, breast, blood, liver) with the maximum lift of recall for the breast and bone cancer (10). For the rest cancer types the lift of recall ranges from 1.33 (for the pancreatic cancer) to 3.33 (for overall cancer profile). The lift of recall for all probability thresholds for these datasets is presented in Fig. 6c.

The variable importance analysis (Fig. 6d) showed that the quadruplex coverage has the positive median coefficient for all cancer types except for the prostate cancer (− 0.001), which is very close to zero in comparison to other types of cancer where the maximum coefficient is observed for the bone and breast cancer (0.64 and 0.62). Analogously, for all cancer types short stem-loops also demonstrate positive relationship with cancer breakpoints hotspots. Only for long stem-loops the relationship is negative in most cases excluding the pancreatic cancer (0.02) with the greatest median coefficient for the brain cancer (− 0.43).

Selection of the strongest predictors for each cancer type leads to the conclusion that for the breast and bone cancer it is the quadruplexes coverage, for the brain cancer – long stem-loops and for the rest of the cancers – short stem-loops.

### Model comparisons

**Fig. 7:**
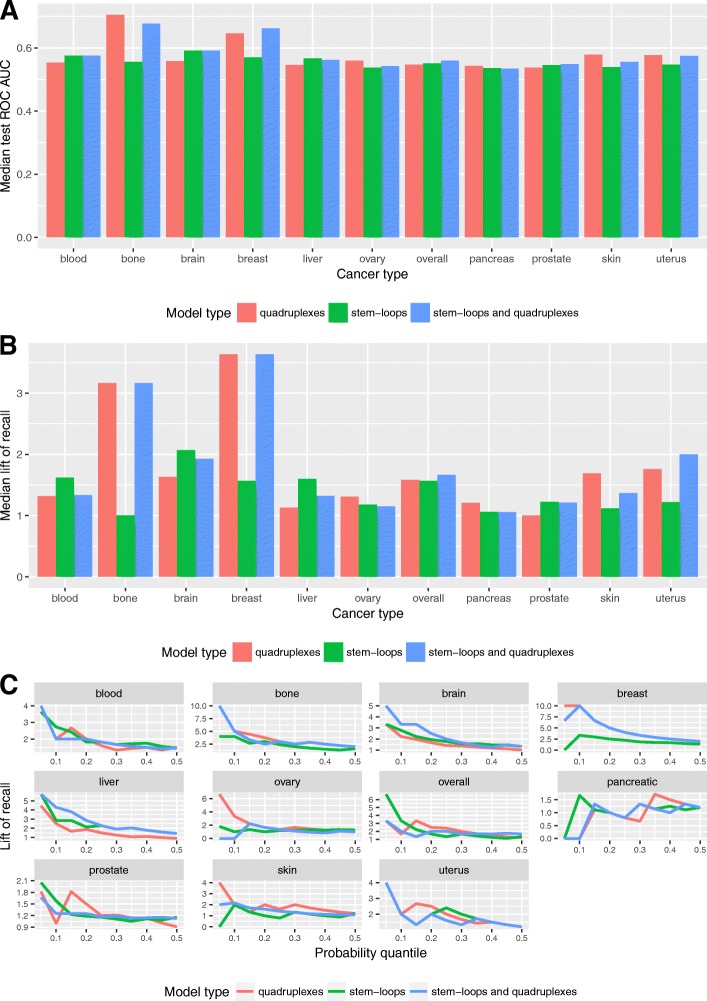
Model comparison. **a** Median test AUC for all cancer types and models. **b** Median lift of recall for all cancer types and models. **c** Lift of recall for all cancer types and models for all probability thresholds for the best models

We built three types of models for 236 datasets of 10 cancer types with 6 aggregation levels and 5 labeling types: stem-loop-based models, quadruplex-based models and joint stem-loop and quadruplex-based models. Additional file 1: Figure S9 summarizes performance of all models for different cancer types concerning the median test AUC and the median recall. It could be seen that for the majority of cancers the difference in the model performance is not significant (except for the breast and bone cancer). Strictly speaking, the analysis of the median test AUC of different models for each cancer type (Fig. 7a) leads to the conclusion that only models for the breast and bone cancer demonstrate relatively high performance in comparison to other cancer types.

At the same time, considering the median lift of recall (Fig. 7b) the difference in model performance is noticeable for some other types of cancer too. In addition, comparing different cancer types, difference in the median test AUC is less significant than in the median lift of recall. For example, median test AUC of the bone cancer is 1.21 times higher than the median test AUC for all cancer types and models. The median lift of recall shows 2.32 growth in comparison to the median lift of recall of all cancer types and models, which is equal to 1.37. This demonstrates that ROC AUC is not very sensitive in cases of imbalanced classes.

In general, according to the median lift of recall quadruplex-based models are higher in performance for the bone, breast, skin, ovary, pancreatic cancer while stem-loop-based models are higher for the blood, brain, liver, and prostate cancer. For the overall cancer profile and uterus cancer the highest performance is achieved by the joint model. It is worth noting that the stem-loop-based and quadruplex-based models demonstrate almost equal prediction power for the overall cancer profile, which incorporates breakpoints density of all cancers.

The Fig. 7c shows the lift of recall for the best models for each cancer type. The results show that for some cancer types one model is better for all probability quantiles (i.e., quadruplex-based model for the breast and bone cancers). For other cancer types the choice of model depends on the probability quantiles.

The model with the highest lift of recall and the model with the highest median lift of recall are different for the blood, brain and overall cancers. At the same time all three best models show the same lift of recall for the uterus cancer. Stem-loop and quadruplex-based models have the same performance only in a few cases: considering the median lift of recall for the best model – for the bone and breast cancer, and considering the lift of recall for the best model – for the bone, breast, blood and liver cancer.

Our results show that adding the second predictor to the best model (stem-loops to quadruplexes or vice versa) in almost all the cases does not improve the model predictive power, but on the contrary could decrease it. For quadruplex-based models with one predictor relative difference between the median train and test AUC ranges from − 4.5% to − 0.9%, for stem-loop-based models with two predictors - from − 3.6 to 2.8% and for the joint stem-loop and quadruplex-based model - from − 2.2 to 4.3%. This variance could be explained by the fact that in the case of extremely imbalanced classes introduction of a weak predictor into a model leads to a fitting noise.

We observed that the tissue type specificity is revealed already at the level of breakpoint hotspots formations. Given the fact that breakpoint hotspots hardly overlap with each other in different types of cancers (see the Jaccard similarity matrix in Additional file 1: Figure S3), the model trained on one type of cancer more likely will not work well for predicting breakpoint hotspots in the other type of cancer. To verify this we took the best model trained to predict hotspots in the blood cancer and applied it to the breast and pancreatic cancers (Additional file 1: Figure S10). Out of 20 combinations of different aggregation levels and labelling types only for 1 combination (500 kB; 0.5%) the blood cancer model showed better results than that of the model trained specifically for the pancreatic cancer and only for 2 combinations (100 kB; 0.1% and 500 kB; 0.1%) the blood cancer model showed better results than that of the breast cancer model. Also, in the majority of the cases tissue-specific models perform better than the model for the generalized cancer profile, which supports the tissue type specificity of breakpoint hotspots formation.

## Discussion

Determination of causes of cancer genome translocations is an active area of research. Cancer genome rearrangements are tightly connected with the DSBs. Many studies have been attempting at finding sequence-based determinants for DSBs and recently machine learning models have been successfully employed for this purpose. Analysis of somatic copy number alteration breakpoints showed that the regions around somatic copy number variants are enriched with quadruplexes and hypomethylated sites [8]. Using DSBcapture data [13] for training, DSBs were predicted with the Random forest model using epigenetic marks, chromatin state and DNA motifs [11]. The most important predictor appeared to be DNAse binding sites followed by CTCF motif and epigenetic marks H3K4me1, H3K4me2, H3K4me3, and H3K27ac [13]. Different types of non-B-DNA structures (Z-DNA, cruciform DNA, G-quadruplexes, R loops and triplexes) have been documented to be a causative agent in translocations of several genes (see [5] for a review).

A number of cases of recurrent chromosomal translocations are implicated with non-B DNA structures. The reported examples when stem-loop structures are causative agents of genome rearrangements include: frequent recurrent translocations in the sperm genomes where the breakpoint occurs within the palindromic AT-rich repeat region [14]; the translocation in the gene NF1 in patients with neurofibromatosis containing palindromic AT-rich repeat region [15]; the recurrently mutated promoter of PLEKHS1 gene that contains an inverted repeat [16]. Quadruplexes are often found in telomeres and promoters and they were also reported to be associated with translocations. Quadruplexes were found in the promoter of c-kit oncogene [17], HOX11 gene [18], and in the fragile regions of near the genes BCL1(CCND1) MTC, E2A(TCF3), BCR, NCOA4, HOX11, ERG, FLI1, TMPRSS2 [19].

Non-B DNA structures are formed in the regions of unwound DNA, which occur in the cell during transcription and replication and can mechanistically induce genome instability [20]. At the same time these structures are located in promoters and near other functional elements and perform important regulatory functions. We see that breakpoint hotspot distributions are specific to cancer types, reflecting the fact that in different tissues different genomic regions are susceptible to damage but the underlying mechanisms should be similar and related to the genome regulation at the tissue-specific level.

The purpose of this study was to compare the impact of stem-loops and quadruplexes on breakpoint hotspots’ formation in different types of cancers as well as to study the variation at the individual level of individual cancer genomes. For that we built machine-learning models predicting breakpoint hotspots based either on stem-loops and/or quadruplex genome-wide coverage. The results of our modeling showed that all cancer types could be divided in two groups – one group is with stem-loop-based models having a higher predictive power (blood, brain, liver, and prostate) and the other is with quadruplex-based models having a higher predictive power (bone, breast, skin, ovary, pancreatic). Characteristically, the joint model built on both stem-loops and quadruplexes did not result in a better performance.

The analysis of machine learning model performance at the level of individual genomes revealed the characteristic trends inherent to various cancer types. In general the variance is higher for stem-loop-based models compared to quadruplex-based models. The highest variance in the median test AUC is observed for the bone cancer both for stem-loop- and quadruplex-based models, and the lowest for the liver, prostate, pancreatic for stem-loop-based models, pancreatic and brain for the qudruplex-based models, and the prostate for the joint models.

Within the boundaries of one cancer type there are genomes for which models achieve a very high performance. Thus, for a breast cancer sample the lift of recall equals to 10 for the quadruplex-based model, while for the same sample the lift of recall is less than one for the stem-loop-based model. And vice versa, a sample from the liver cancer has the lift of recall of 5.71 for the stem-loop-based model and it equals to 2.85 for the quadruplex-based model.

There are three types of cancer for which the performance of the quadruplex-based model is considerably better than that of the stem-loop model (0.1–0.3 in the median AUC and 3–4 in the lift of recall): bone (median AUC = 0.86, lift of recall 10), breast (median AUC = 0.94, lift of recall 10) and ovary (median AUC = 0.68, lift of recall 6.7) cancer. For the prostate and brain cancers stem-loop models have better performance than quadruplex-based models. For uterus and pancreatic cancer two types of models have almost equal prediction abilities.

For stem-loops we studied three ranges of different size as they can potentially be important in different genomic processes. Thus, we found that short (stem 6–15 bp) and medium (stem 15–30 bp) stem-loops have more correlation with breakpoint hotspots rather than long (stem 16–50 bp) stem-loops. We excluded medium size stem-loops from the modeling because short and medium stem-loops are 94%-correlated. The impact of short stem-loops is positive while the impact of long stem-loops is negative. This finding supports the idea that short stem-loops are likely to be formed during transcription or replication processes with DNA being in a single-stranded state.

We also checked how known translocations (from Mitelman Database of Chromosome Aberrations in Cancer [21]) overlap with the defined hotspots (Additional file 1: Figure S11 and Additional file 3). Since a hotspot is a region of a high breakpoint density with the length from 10 kb to 1 Mb, well-known translocations leading to recurrent gene fusions are not necessarily lie in the regions of high breakpoint density; the density of the region harboring these translocations can be moderate or even low. We found 5 known gene fusions that fall into breakpoint hotspots (namely, IGL-CCND1, CTNNB1-PLAG1, FUS-ATF1, IGH-CCND1, KMT2A-AFF1). The other known translocations including IGH-MYC, IGH-BCL2, and others are not in the hotspot regions, or regions of significantly high breakpoint densities compared to the other genomic regions. This could be explained by the fact that different mechanisms are responsible for the formation of dense breakpoint regions and recurrent point translocations leading to gene fusions. Totally, from 1273 analyzed translocations 362 fall into hotspots (Additional file 3).

All the models detected false positives (Additional file 2), which is the number of genomic regions designated by a model as breakpoint hotspots but they are not found in the real data. These false positives could be considered as genomic regions similar to breakpoints hotspots by the DNA secondary structures’ coverage, and thus can be areas of potential genome breakage.

Since breakpoint hotspots are poorly correlated between different cancer types, the difference in contribution of stem-loops and quadruplexes are tissue-specific. As it was mentioned earlier machine learning modeling with inclusion of epigenetic information revealed that epigenetic factors such as DNAse biding sites, some histone modifications and methylation states are important predictors. The interrelation of non-B DNA regulatory structures with epigenetic regulation is a poorly studied area. Our results suggest that tissue-specific impact of stem-loops and quadruplexes most likely reflects the difference in non-B DNA structure tissue-specific regulation – the area that has not yet been extensively studied and is a subject for future research.

## Conclusions

Using machine learning approach, we performed the comprehensive analysis of cancer breakpoint hotspots from 2234 samples of 10 cancer types available at ICGC with the aim to study the impact of stem-loops and quadruplexes on cancer breakpoint hotspot formation and found that stem-loops are important determinants for the blood, brain, liver, and prostate cancer while quadruplexes - for the bone, breast, ovary, pancreatic, and skin cancer. For specific datasets models showed very high prediction accuracy. Cancer genomes are highly heterogeneous, and this heterogeneity is also manifested at breakpoint hotspots distribution and non-B structures contribution to mutagenesis. From one hand, non-B structures are important regulatory functional elements, from the other they cause chromosome instability. Non-B DNA structures’ contribution to mutagenesis is defined by the regions of chromosomal activity. The role of non-B structures as functional regulatory elements at the tissue-specific level are yet to be discovered at the genome-wide scale.

## Methods

### Data

Data of cancer breakpoints were downloaded from the International Cancer Genome Consortium Data Portal (release 25). The fields of the data table used in the research are presented in (Additional file 1: Table S9). In total, the available data covers 10 cancer types and 2234 samples.

### Breakpoints selection

As it was mentioned in data fields description, there are two columns – «chr_from_range» and «chr_to_range» – which show the radius in base pairs around breakpoint position stated at «chr_from_bkpt» and «chr_to_bkpt» which could contain real breakpoint. This way these fields demonstrate inaccuracy in breakpoint location definition. During checking the distributions of these fields it was noted that there are missing values which means that the quality of measurement is perfect and fields «chr_from_bkpt» and «chr_to_bkpt» give precise location of breakpoint (Additional file 1: Figure S12). Taking this into account these missing values should be replaced with 0.

Although «confidence intervals» («chr_from/to_range») around breakpoints in most cases are narrow, there are some outliers which will bring the noise to data. Besides it could be seen that 95% of breakpoints have a range not greater than 10 in both cases (for donor chromosome as well as acceptor chromosome) so this value is used as a threshold.

The list of all breakpoints was formed where each breakpoint is characterized by chromosome, position, range and cancer type. Then breakpoints with range higher than 10 were excluded from consideration. For each of the rest breakpoints the beginning and the end of breakpoint were calculated accounting for range.

### Density calculations

For each cancer type genome was split on disjoint “windows” of specified length (10 kb, 100 kb, 1 Mb, etc.) and for each window breakpoints density was calculated as the number of breakpoints located in a window divided by the total number of breakpoints in the genome.

### General Cancer profile calculations

The general cancer breakpoint density profile was calculated using Bayes formula of total probability. Let A be an event of a breakpoint occurrence in a given window. P(A|B_i_) – is the probability of breakpoint occurrence in a given window for a specific cancer type B_i_, which is the calculated density for each cancer type. The total probability of breakpoints for all cancer types can be calculated with the formula P(A) =  ∑ P(A|B_i_) ∗ P(B_i_), where P(B_i_) - probability of a specific type of cancer. We used the World Cancer Research Fund International (http://www.wcrf.org), which provided the data about the number of new cancer cases in 2012 for each type of cancer (Additional file 1: Table S10). Bone cancer was not presented in the dataset, and we imputed this value with the minimum available value in the source dataset.

### Breakpoint hotspots

We selected high-density regions based on five different quantile thresholds: 1, 0.5, 0.1, 0.05, 0.01%. Number of breakpoints hotspots by cancer type for 6 different length of window (10, 20, 50, 100, 500 kb and 1 Mb) and for 5 different thresholds is given in the Additional file 1: Table S2. The table demonstrates that cancer profiles have very small (less than 10) number of breakpoints hotspots for some labeling types at aggregation levels of 50 kb, 100 kb, 500 kb and 1 mb. This number of breakpoints hotspots (or positive examples) is not enough for building machine learning models that’s why these profiles were excluded. Besides there are identical cancer profiles for given aggregation level and cancer type as different “neighboring” labeling types (for example, 0.5 and 1%) give the same breakpoints hotspots locations (17 profiles have a copy in total). Finally, there are 236 cancer profiles for analysis. Breakpoint hotspots at 6 aggregation levels are available in Additional file 4.

### DNA secondary structures annotations and coverage

Human genome annotations with stem-loops (hg19) were downloaded from the DNA punctuation project (http://www.dnapuncutation.org). Labels of three types of stem-loops are available: length of stem: 6–15, length of loop: 0–10, 1 mismatch is allowed (S6–15); length of stem: 15–30, length of loop: 0–10, 5 mismatches are allowed (S15–30); length of stem: 16–50, length of loop: 0–10, 3 mismatches are allowed (S16–50). Annotation of human genome (hg19) with G-quadruplexes was done by applying regular expression [22].

We used coverage as a measure secondary structure density in a given window. For DNA secondary structures of a specified type the coverage in a given window was calculated as the total length of all structures in the window (without overlaps) divided by the window size. Stem-loop coverage at 6 aggregation levels are available in Additional file 5. Quadruplex coverage at 6 aggregation levels are available in Additional file 6.

### Machine learning (ML) model building and evaluation

We performed 15-times repeated 3-fold cross-validation based on the logistic regression with oversampling. The following algorithm was applied to all datasets. Each dataset represents a dataset with the target (0/1 breakpoint hotspots labeling) and predictors (stem-loops and/or quadruplexes coverage, standardized with z-score transformation). 15-times repeated 3-fold cross-validation procedure was applied to each dataset to estimate train and test ROC AUC and model coefficients. In 3-fold cross validation each dataset was split into 3 folds with stratification. One fold was used as a test set and the rest two folds as a train set. Oversampling was done on the train set so that the number of positive examples will be equal to the number of negative examples. Logistic regression was built on the train set.

Recall was calculated on the test set at different probability thresholds. Set of thresholds is defined as thresholds related to specific predicted probability quantiles (0.5, 0.55, 0.60,0.65, 0.70, 0.75, 0.80, 0.85, 0.90, 0.95). Thus, 10% probability threshold will mark 10% of the test set observations with maximal probability as “1” (breakpoint hotspot) and the rest as “0”. The procedure was repeated 15 times and the mean performance based on performance of all built models was used to estimate the model prediction power: the median train ROC AUC, median test ROC AUC point estimate, confidence interval for the mean test ROC AUC based on the standard deviation and standard error, the median coefficient of each predictor, a distribution of the recall and lift of recall on a test set at different probability quantiles.

### Confidence interval calculation

Due to AUC variability caused by the class imbalance it was required to estimate the confidence interval for the mean test AUC. The first type of confidence interval was calculated based on t-interval. As described in the procedure above 15 * 3 = 45 AUC values were generated, its distribution tends to normal according to the central limit theorem and, hence, the t-statistic could be used to calculate confidence interval:

$$ \overset{\sim }{\mathrm{AUC}}\pm {\mathrm{t}}_{1-\upalpha /2}\frac{\mathrm{s}}{\sqrt{\mathrm{n}}} $$

where *n* is the population size, *s* is the standard deviation and t1-alpha/2 is a critical value from the t-distribution.

The second type of confidence interval was calculated based on standard error.

The standard error of the mean test AUC measures the dispersion of sample means around the population mean. As it was shown, the area under ROC curve evaluates the same quantity as Wilcoxon statistics [23] so that its statistical properties (including the standard error) could be used for AUC as well. We used the following formula for SE(W):

$$ \mathrm{SE}\left(\mathrm{W}\right)=\sqrt{\frac{\mathrm{Q}\left(1-\mathrm{Q}\right)+\left({\mathrm{n}}_1-1\right)\left({\mathrm{Q}}_1-{\mathrm{Q}}^2\right)+\left({\mathrm{n}}_0-1\right)\ast \left({\mathrm{Q}}_2-{\mathrm{Q}}^2\right)}{{\mathrm{n}}_0{\mathrm{n}}_1}}, $$,

where *n*_0_*-* number of examples of “normal” class*, n*_1_ - number of examples of “abnormal” class, *Q –* estimate of *AUC*, ${Q}_1=\frac{Q}{{\left(2\kern1em Q\right)}^{,}}{Q}_Z=\frac{2{Q}^Z}{\left(1+Q\right)}$

New confidence interval could be calculated using this standard error estimate of the mean AUC. The model is considered as having prediction power if both confidence intervals do not include 0.5.

### Calculation of the lift of recall

If there is no relationship, the expected median recall of the model should be close to the recall of a random selection while in the random selection taking n% of the data gives approximately n% recall. The metric “lift of recall” can provide an estimate of how the model behaves in comparison to a random. The lift of recall is calculated as the ratio of the median recall to the probability quantile and it measures how the performance of the model differs from a random selection. It is less than 1 in the case of the model’s performance near or worse than a random model and is greater than 1 in the case of the model’s performance better than random.

### Choice of resampling schemes

In order to choose best resampling methods we tested three several resampling schemes: LOOCV, train-test splits and repeated 3-fold cross-validation. LOOCV takes a single point from the data for the validation, and the remaining records are used as the training set. This is repeated as many times as the number of records in the data so that each point is used once for the validation. In the train-test splits we created 100 random splits of the data on the train and test sets separately for each class in proportion of 50/50. In the repeated 3-fold cross-validation a dataset was randomly split in 3 folds separately for negative and positive class. For each split 3 models were trained with oversampling for training data. The procedure repeated 15 times and totally 45 model performance metrics were obtained. Estimation of the model performance was done with F-score (the harmonic mean of the precision and recall).

As a test data set we choose data from the breast cancer with 500 kb aggregation level and 0.5% labeling threshold and only stem-loops as predictors. In the choice of resampling scheme, we chose oversampling as a class balancing method.

### Class balancing techniques

We tested three class balancing techniques, which can affect model performance: oversampling, stratification, and SMOTE. In oversampling we duplicated minority class examples (hotspots, in our case) and made the proportion of two classes equal. In SMOTE we tested 2000, 5000 and 10,000 of oversampling percentages. In stratification method we doubled the size of the imbalanced class (hotspots) and randomly selected the same number of samples from the negative class.

## Additional files

Additional file 1: This file includes supplemental figures (**Figures S1-S11**) and tables (**Tables S1-S10**). (PDF 853 kb)

Additional file 2: Performance metrics for 708 ML models for all cancer types and all aggregation levels and labeling types. (XLSX 138 kb)

Additional file 3: List of 363 translocations form the Mitelman database that fell into hotspots regions. (XLSX 38 kb)

Additional file 4: Breakpoint density at 6 aggregation levels. (XLSX 75829 kb)

Additional file 5: Stem-loop coverage at 6 aggregation levels. (XLSX 46355 kb)

Additional file 6: Quadruplex coverage at 6 aggregation levels. (XLSX 20868 kb)

## Abbreviations

3′ UTR: 3′ untranslated region
5′ UTR: 5′ untranslated region
AUC: Area under the curve
DSB: Double-strand break
G4: Quadruplexes
ICGC: International Cancer Genome Consortium
LOOCV: Leave-one-out cross validation
ML: Machine learning
ROC AUC: Area under the receiver operating characteristic curve
ROC: Receiver operating characteristic
SMOTE: Synthetic minority oversampling technique
