# Supplementary material for: Tissue-specific impact of stem-loops and quadruplexes on cancer breakpoints formation
Source: BMC Cancer. 2019 May 10;19:434. doi: 10.1186/s12885-019-5653-x (PMC6511154; doi:10.1186/s12885-019-5653-x)
Supplement: Supplementary file 1 — This file includes supplemental figures (Figures S1-S11) and tables (Tables S1-S10). (PDF 853 kb) [file 12885_2019_5653_MOESM1_ESM.pdf]

Supplementary figures

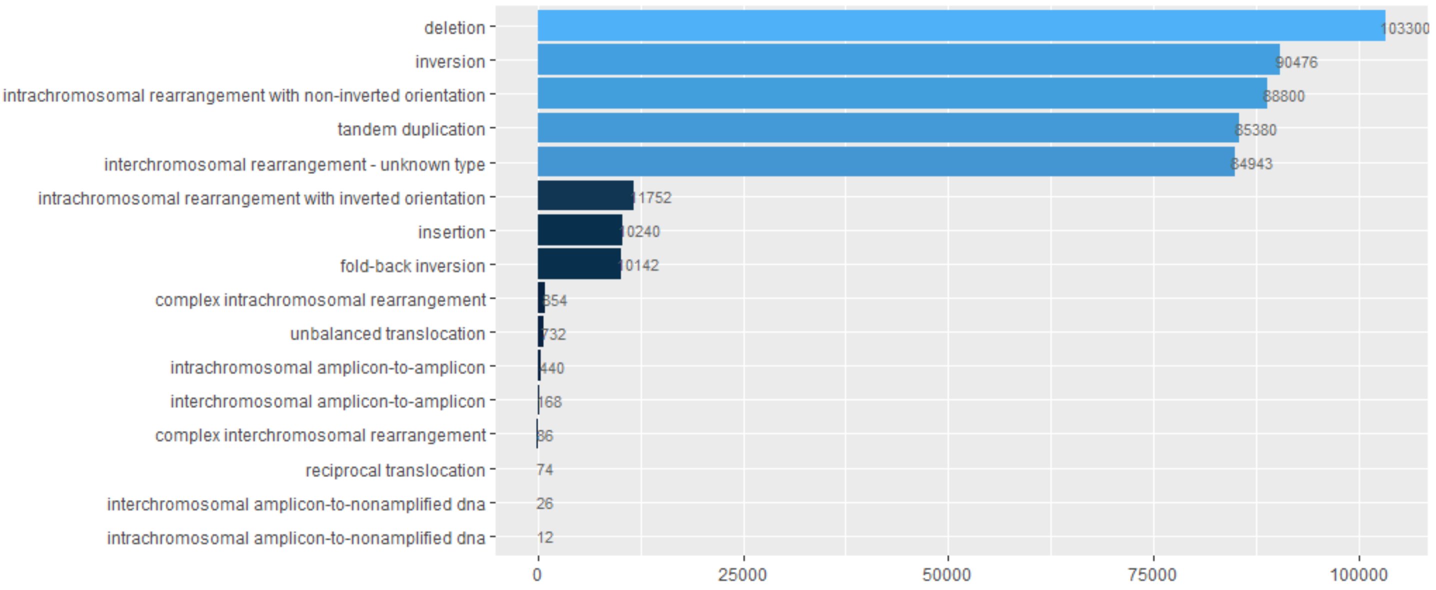

Figure S1. The number of breakpoints by variant type.

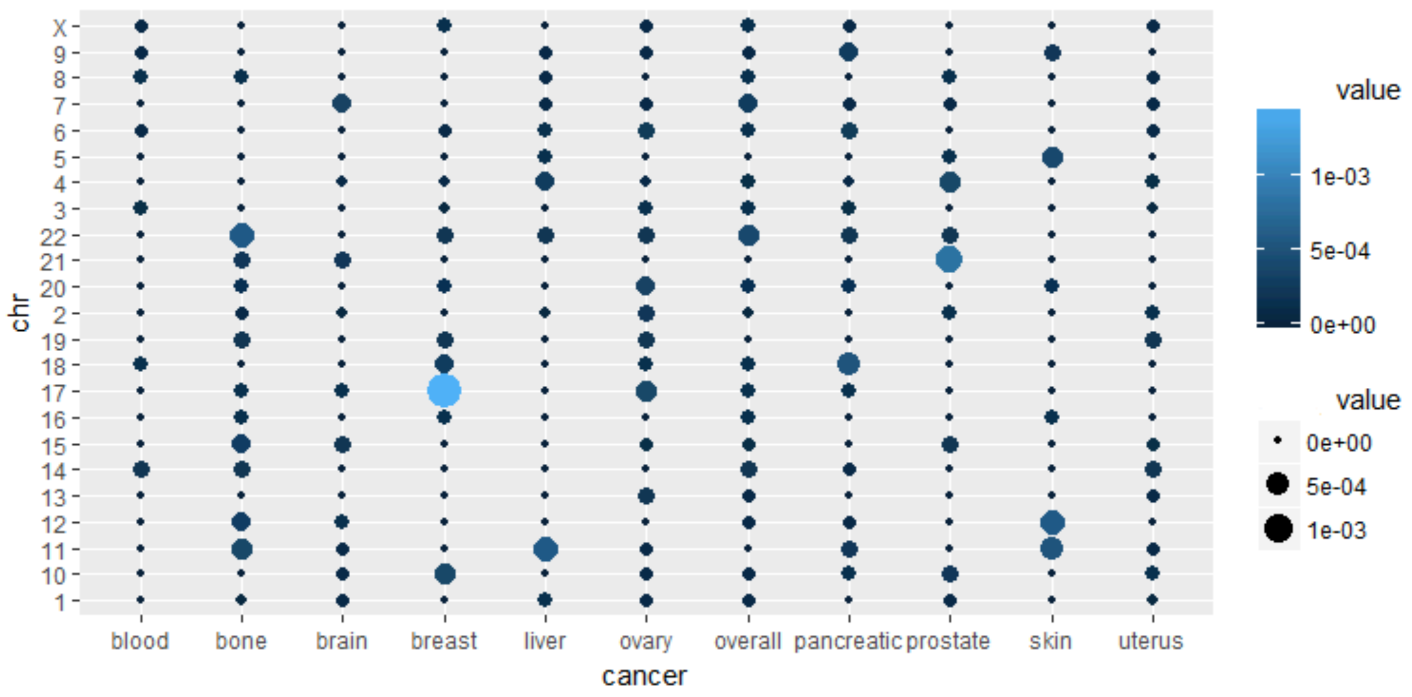

Figure S2. The number of breakpoint hotspots by the chromosomes and types of cancer for 0.01% labeling type.

A

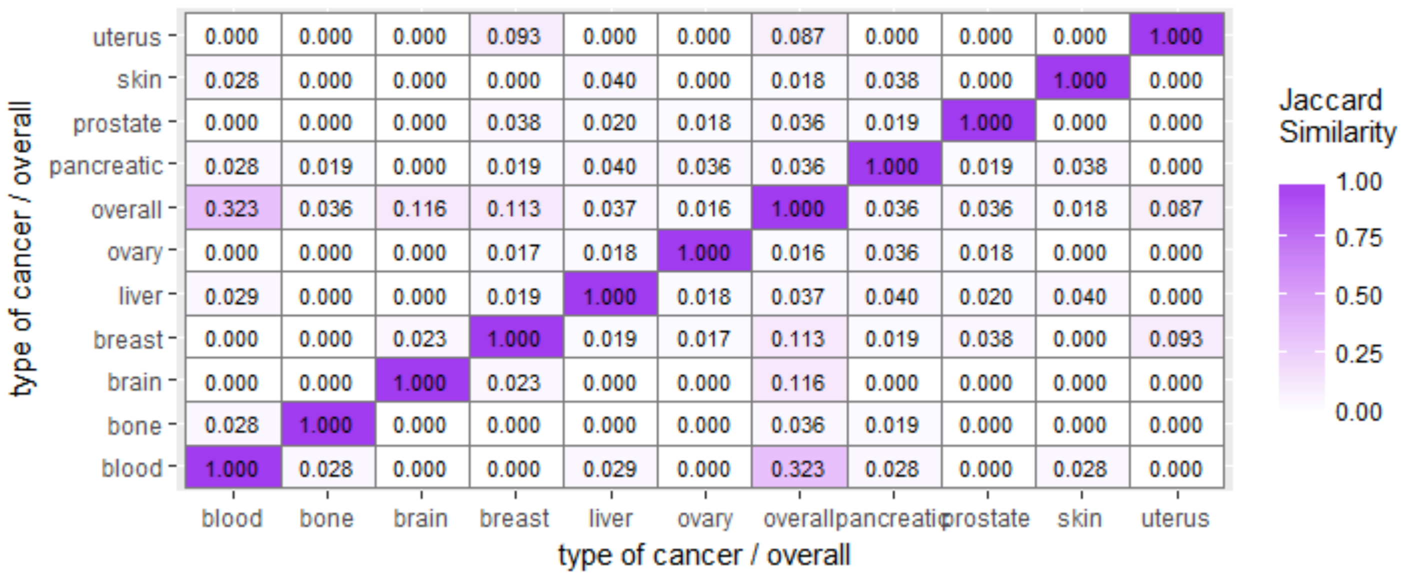

B

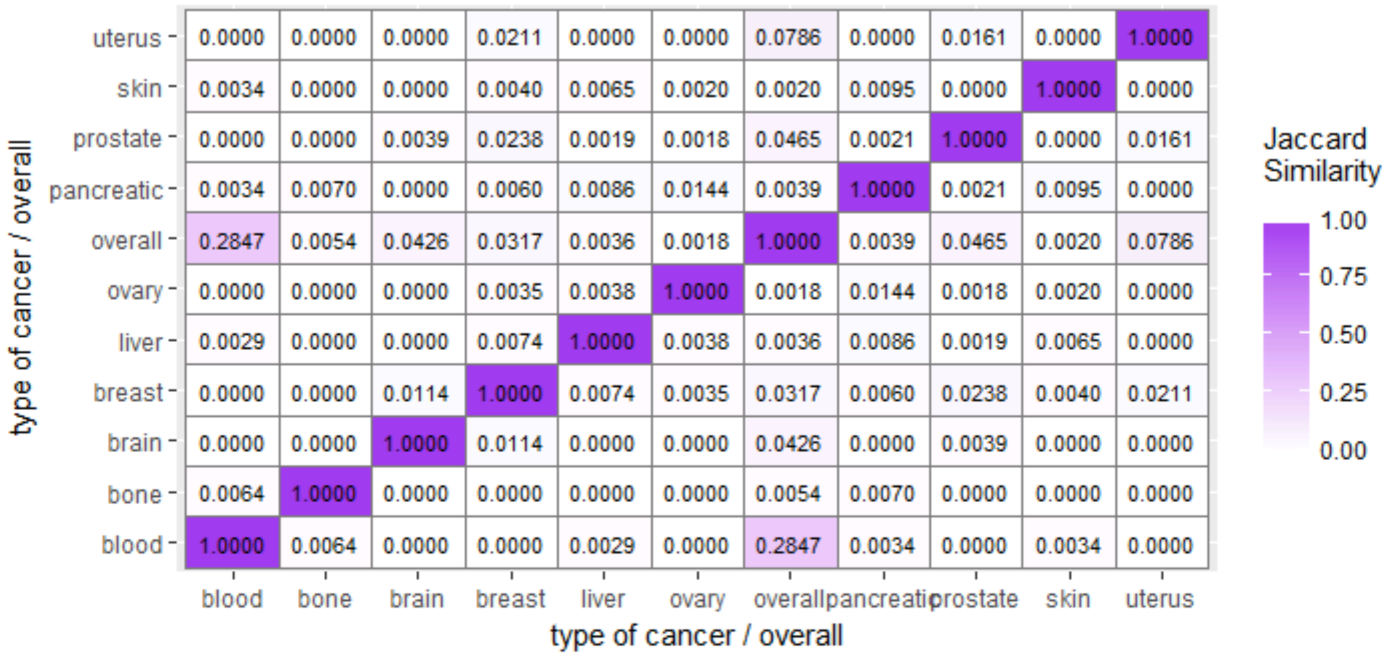

Figure S3. A. Jaccard similarity between breakpoints hotspots profiles for different cancer types at 0.1% labeling type. **B.** Jaccard similarity between breakpoints hotspots profiles for different cancer types at 0.01% labeling type.

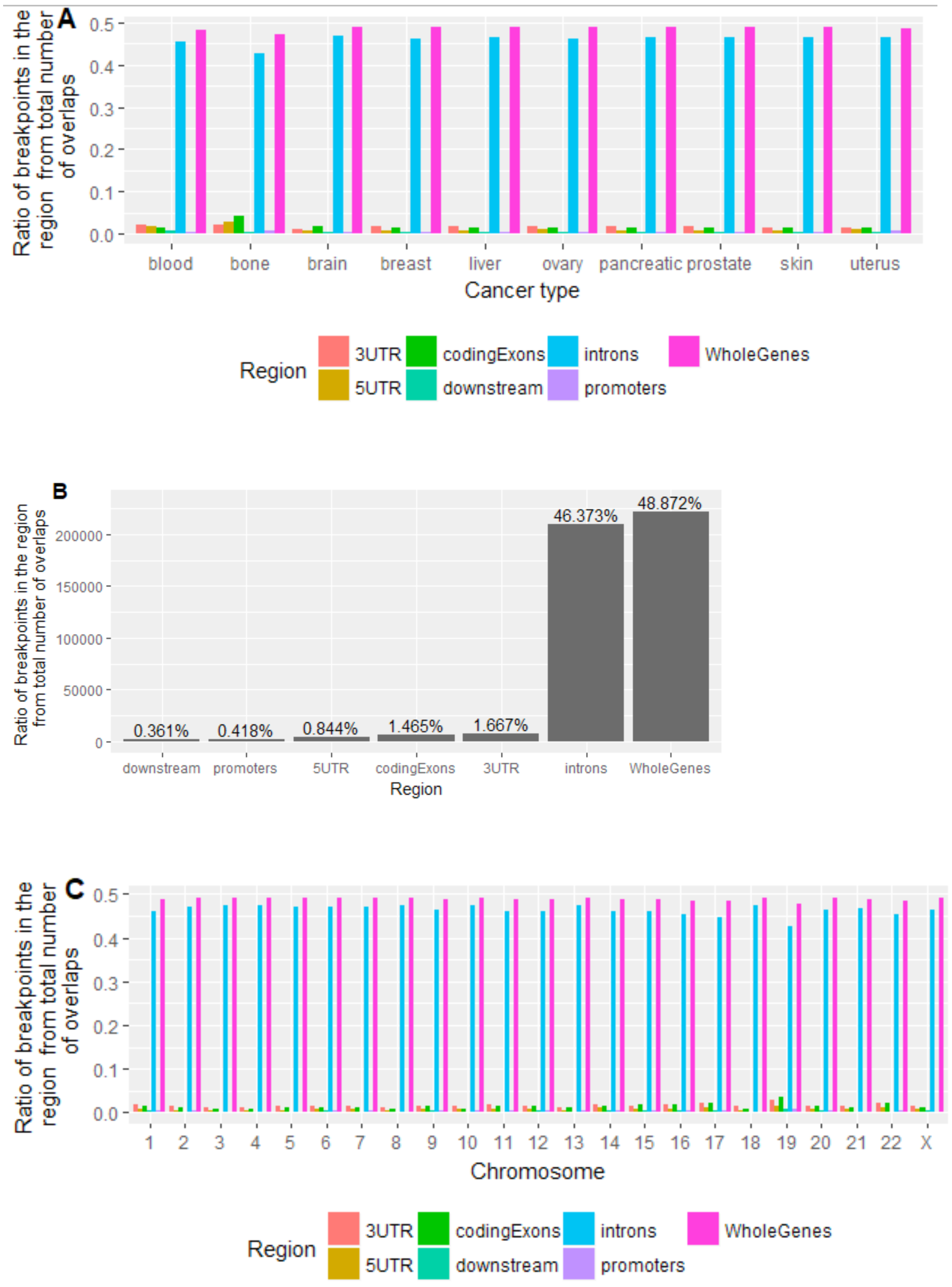

Figure S4. A. Distributions of breakpoints intersecting with whole genes, exons, introns, 5'UTR, 3'UTR, promoters and downstream regions in different cancers. **B.** Distributions of the total number of breakpoints over genomic regions. **C.** Distributions of the total number of breakpoints over genomic regions per chromosomes.

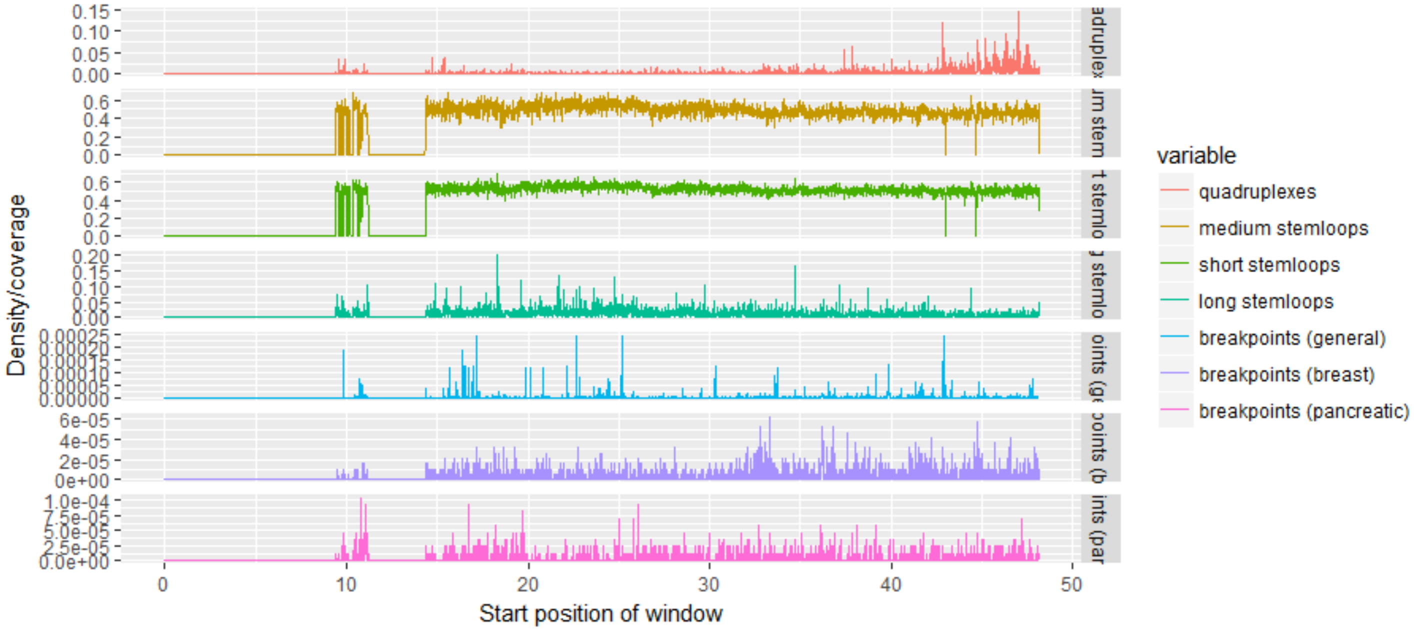

Figure S5. Breakpoints density and coverage of stem-loops and quadruplexes for 21 chromosome (in Mb).

A

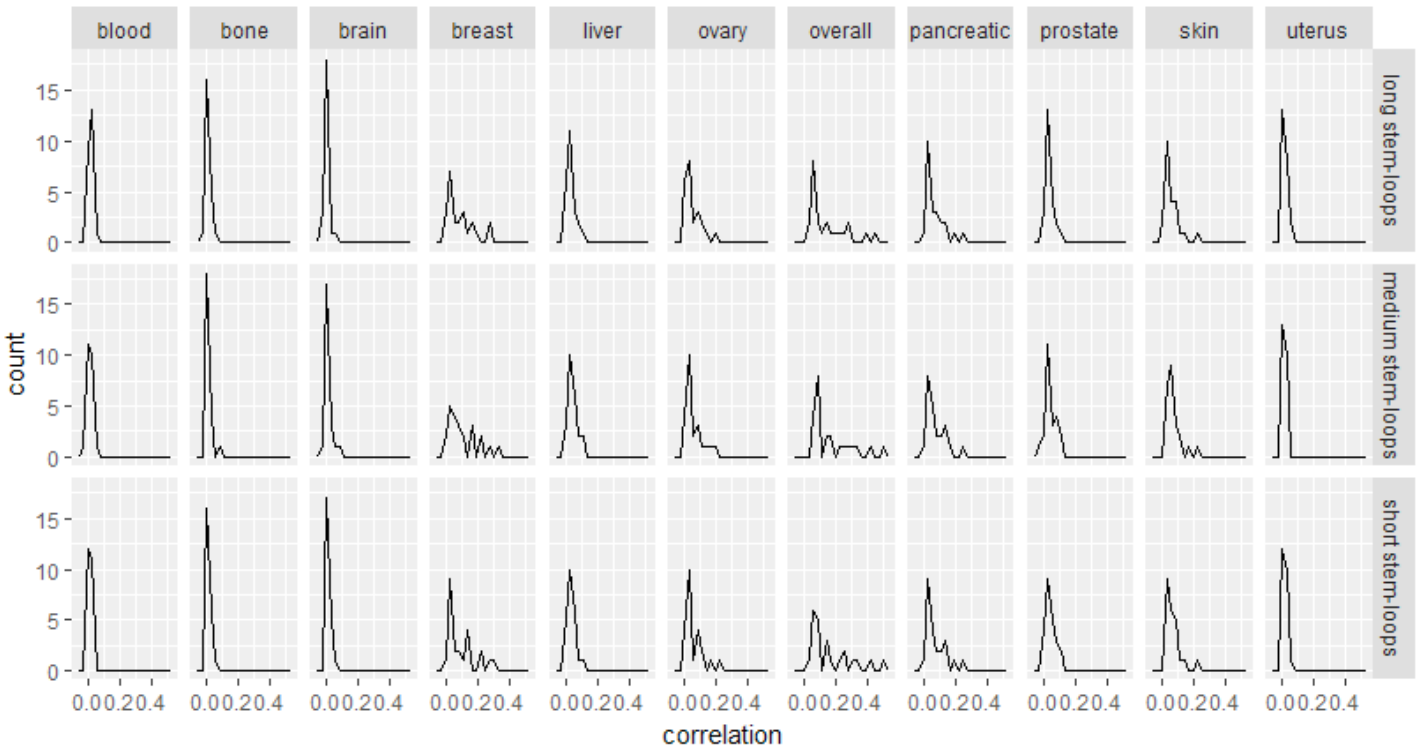

B

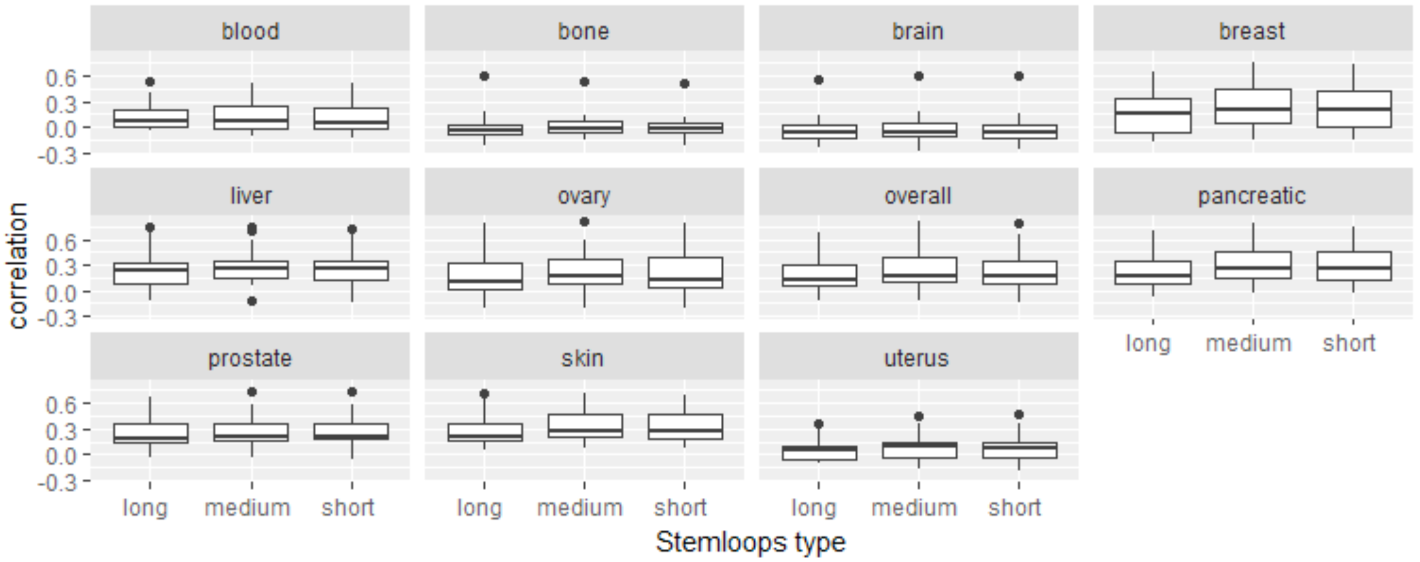

Figure S6. A. Histograms of chromosomes' correlations between densities of cancer profiles and the coverage of stem-loops at 10 kb aggregation level. **B.** Boxplots of chromosomes' correlations between densities of cancer profiles and the coverage of stem-loops at 1 Mb aggregation level.

A

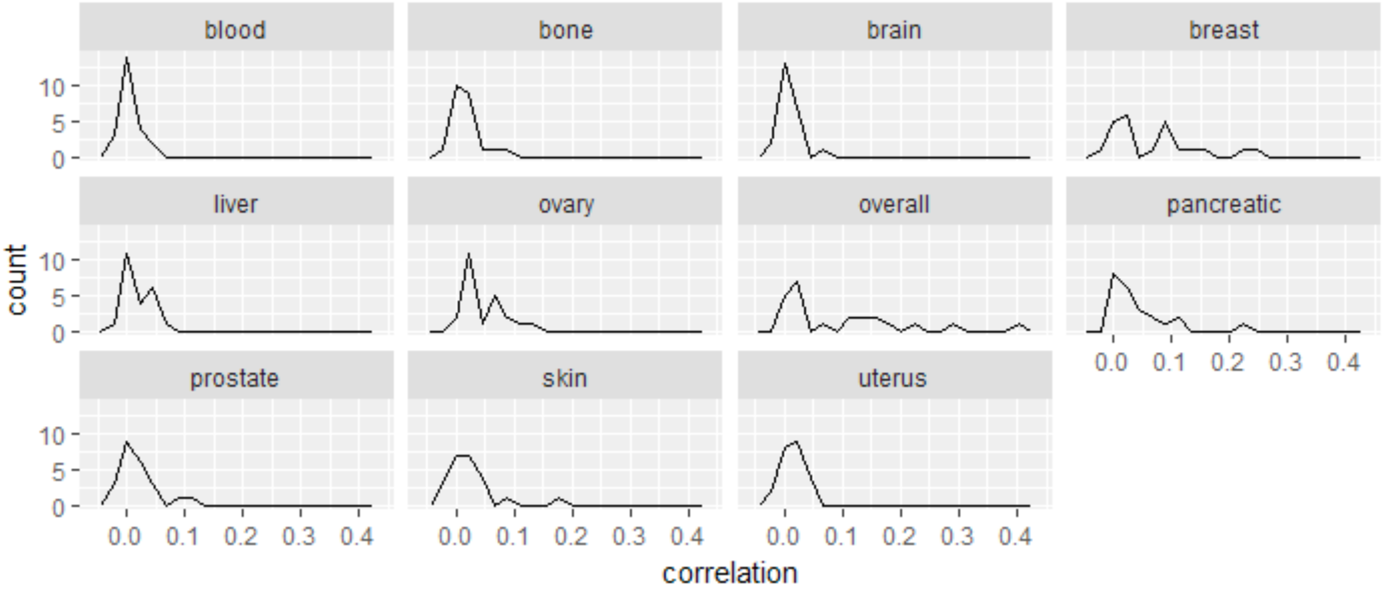

B

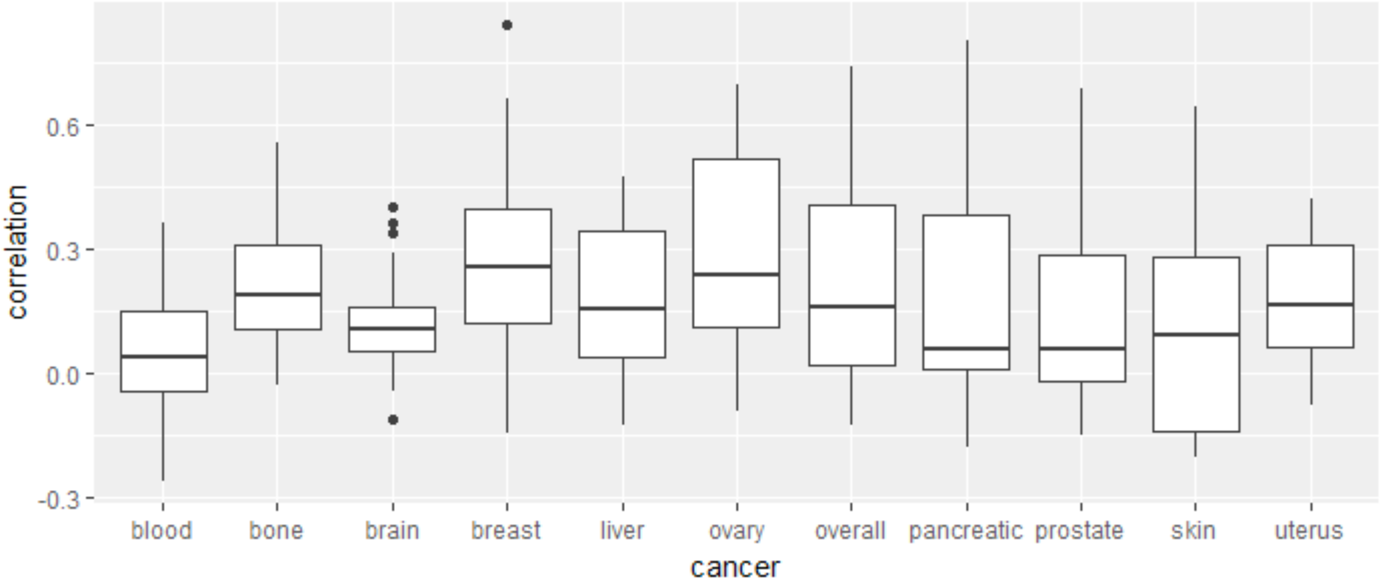

Figure S7. A. Histograms of chromosomes' correlations between the densities of cancer profiles and the coverage of quadruplexes at 10 kb aggregation level. **B.** Boxplots of chromosomes' correlation between the densities of cancer profiles and the coverage of quadruplexes at 1 mb aggregation level.

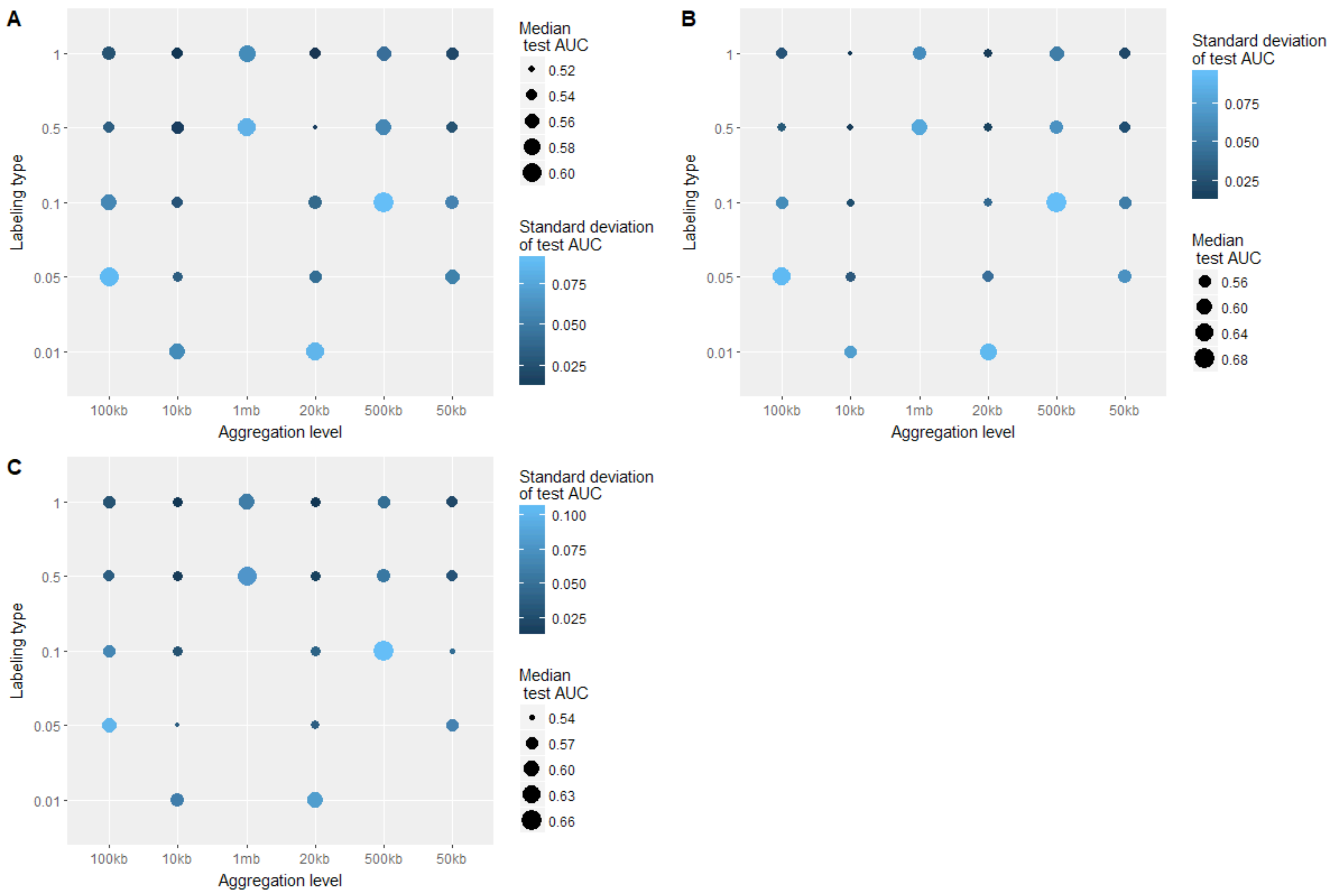

Figure S8. Distributions of the median value of the standard deviation in datasets and the median of test AUC grouped by the aggregation level and the labeling type. **A.** Stem-loop-based models. **B.** Quadruplex-based models. **C.** Joint stem-loop and quadruplex-based model.

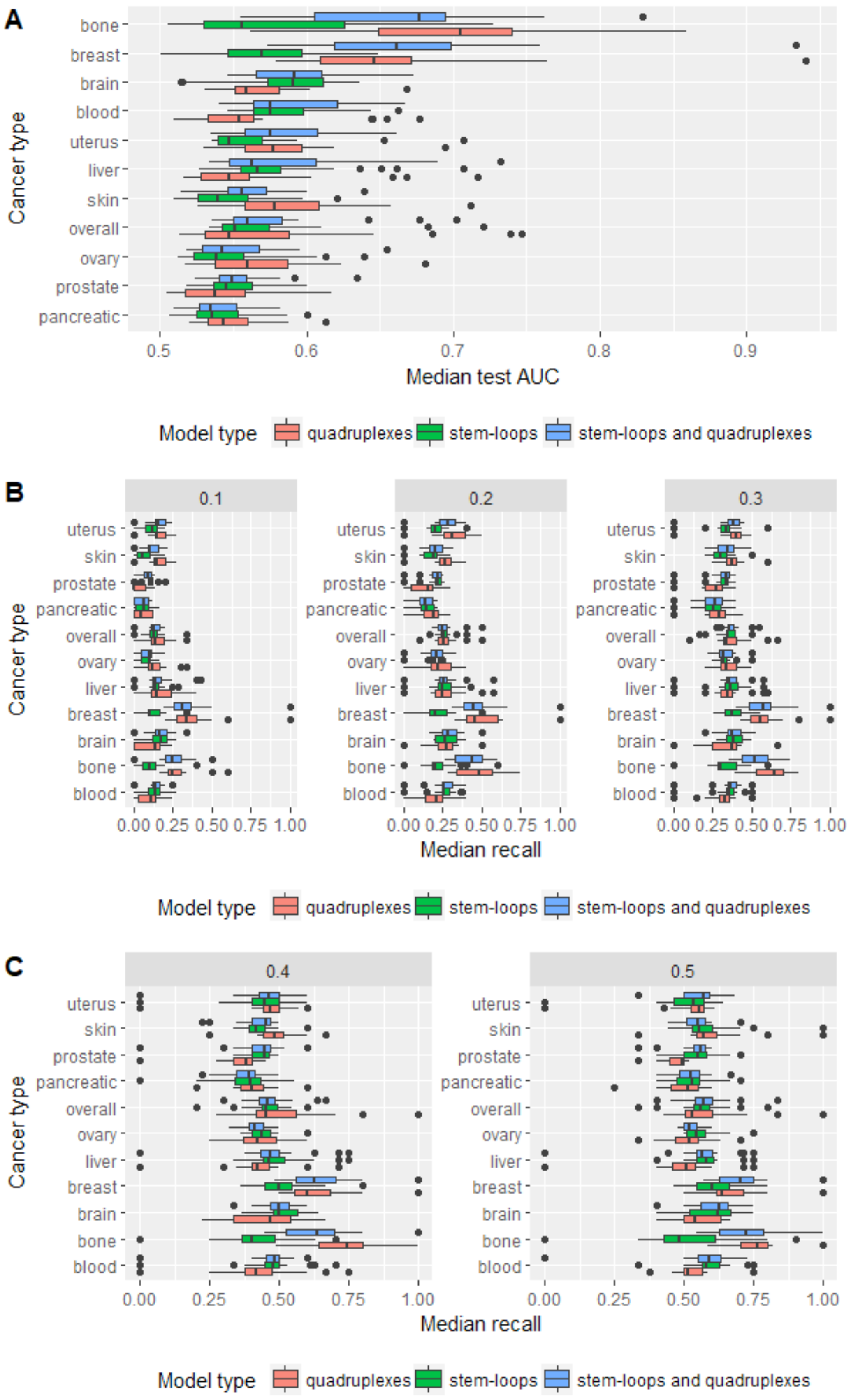
A
bone
breast
brain
blood
uterus
liver
skin
overall
ovary
prostate
pancreatic
Cancer type
0.5
0.6
0.7
0.8
0.9
Median test AUC
Model type
quadruplexes
stem-loops
stem-loops and quadruplexes
B
0.1
0.2
0.3
uterus
skin
prostate
pancreatic
overall
ovary
liver
breast
brain
bone
blood
Cancer type
0.00
0.25
0.50
0.75
1.00
Median recall
Model type
quadruplexes
stem-loops
stem-loops and quadruplexes
C
0.4
0.5
uterus
skin
prostate
pancreatic
overall
ovary
liver
breast
brain
bone
blood
Cancer type
0.00
0.25
0.50
0.75
1.00
Median recall
Model type
quadruplexes
stem-loops
stem-loops and quadruplexes

Figure S9. Comparison of three models – stem-loop-, quadruplex- and joint stem-loop and quadruplex-based models. **A.** Median Test AUC. **B-C.** Median recall for 5 probability quantiles: 0.1, 0.2, 0.3, 0.4, 0.5.

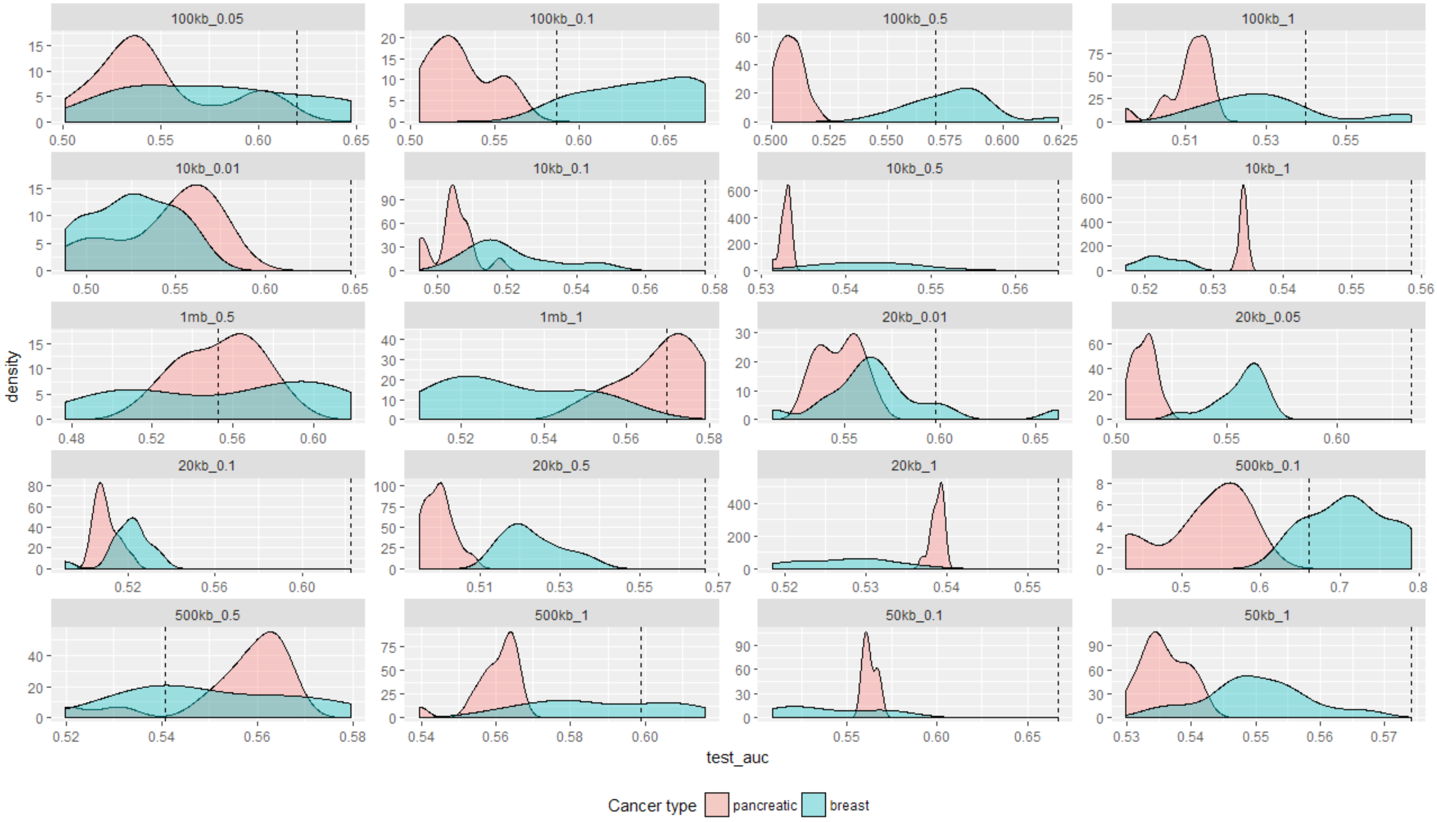

Figure S10. Prediction by the best blood cancer model the breakpoint hotspots in the breast and pancreatic cancers. All combinations of hotspot regions and labeling types are considered. The dashed line designates the median test AUC achieved by the best blood cancer model to predict hotspots in the blood cancer.

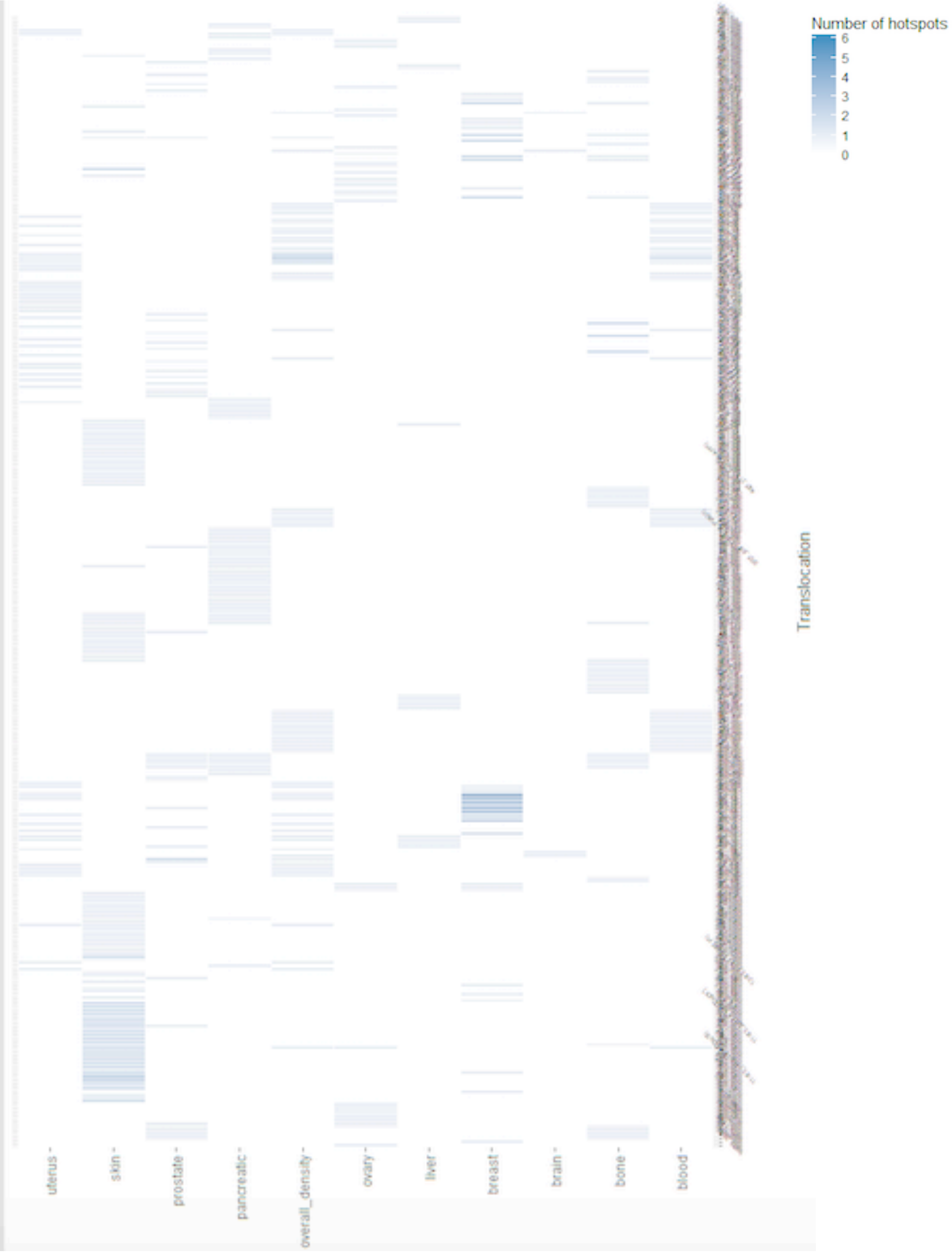
Number of hotspots
6
5
4
3
2
1
0
Translocation
uterus
skin
prostate
pancreatic
overall_density
ovary
liver
breast
brain
bone
blood

Figure S11. Intersections of breakpoint hotspots with known translocations from the Mitelman database over the entire genome in different cancers. 5 known gene fusions IGL-CCND1, CTNNB1-PLAG1, FUS-ATF1, IGH-CCND1, KMT2A-AFF1 re adesignated.

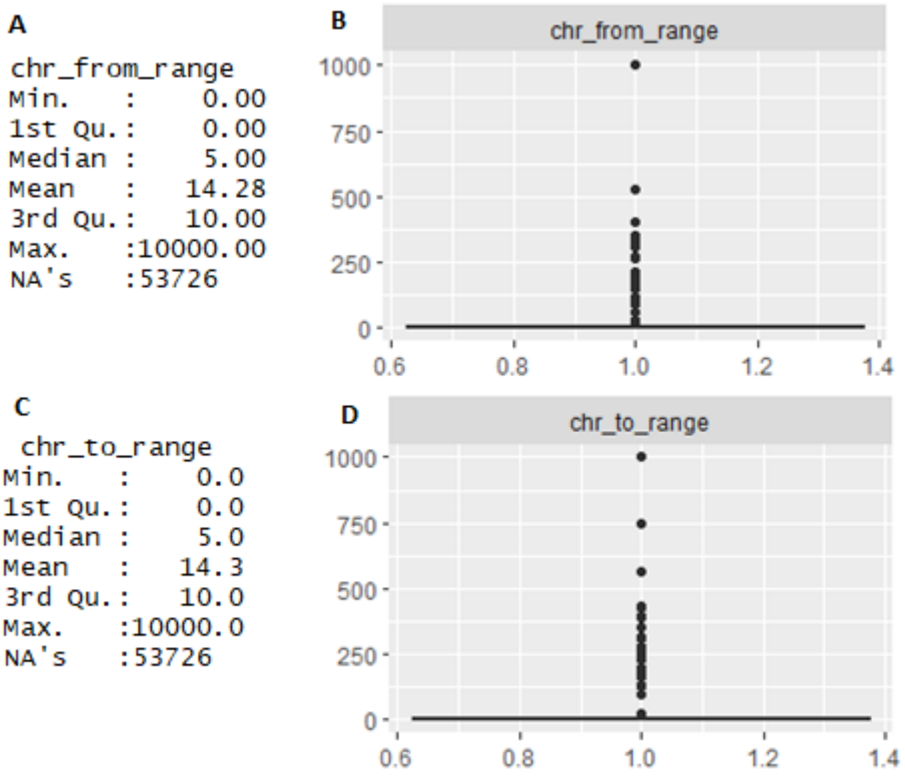

Figure S12. Boxplots of the fields "chr_from_range" and “chr_to_range”.

Supplementary tables

Table S1. The number of samples, donors and breakpoints grouped by cancer type.

Cancer type	Number of samples	Number of donors	Number of breakpoints
brain	72	72	1564
blood	118	118	2330
bone	117	117	2546
uterus	16	7	6782
liver	255	243	22324
prostate	212	161	48126
skin	190	190	54688
ovary	115	93	71446
pancreatic	495	447	85769
breast	644	644	191850

Table S2. The number of breakpoint hotspots grouped by the cancer type, aggregation level and labeling type.

Cancer type / Aggregation level	Labeling type	blood	bone	brain	breast	liver	ovary	overall	pancreatic	prostate	skin	uterus
10 kb	0.01	10	27	17	28	25	31	31	27	27	27	19
	0.05	84	74	43	152	86	74	152	74	138	121	48
	0.1	84	74	243	291	256	277	295	215	268	212	48
	0.5	622	1427	1088	1251	690	1024	1519	1404	1443	732	564
	1	1533	1427	1088	1919	690	1024	3023	1404	2689	1603	2276
20 kb	0.01	14	11	12	16	14	10	16	14	16	11	5
	0.05	67	46	53	70	67	54	74	69	69	68	71
	0.1	97	84	53	138	67	132	152	141	115	147	71
	0.5	634	624	546	698	676	483	760	695	619	667	740
	1	1489	1370	1027	1389	1081	1369	1519	1394	1461	1312	740
50 kb	0.01	5	6	6	7	7	5	7	6	7	7	5
	0.05	31	29	16	30	21	29	31	27	31	30	31
	0.1	31	33	42	59	60	50	61	48	54	60	41
	0.5	170	106	72	288	209	279	304	245	278	287	149
	1	170	606	335	577	363	419	608	432	533	564	149
100 kb	0.01	3	4	4	4	4	4	4	4	4	2	3
	0.05	11	15	10	16	14	13	16	16	16	15	16
	0.1	25	29	26	31	25	28	31	29	30	30	21
	0.5	144	122	96	146	136	123	152	142	144	152	127
	1	232	122	96	297	250	231	304	301	281	304	265
500 kb	0.01	1	1	1	1	1	1	1	1	1	1	1
	0.05	4	4	4	4	4	4	4	4	4	4	2
	0.1	7	7	7	7	7	7	7	7	7	7	7
	0.5	25	29	26	31	29	30	31	29	31	31	21
	1	48	56	44	61	51	58	61	58	61	58	44
1 mb	0.01	1	1	1	1	1	1	1	1	1	1	1
	0.05	2	2	2	2	2	1	2	2	2	2	2
	0.1	4	4	4	4	4	3	4	4	4	4	2
	0.5	13	13	14	16	16	16	16	16	16	15	15
	1	23	29	25	31	31	31	31	31	30	31	31

Table S3. The stem-loops based models: the median and the third quantile of recall (in brackets) for each cancer type and quantile.

	Probability quantiles				
Cancer type	0.1	0.2	0.3	0.4	0.5
blood	0.1345<br>(0.1685)	0.2571<br>(0.2904)	0.3567<br>(0.3812)	0.4762<br>(0.5000)	0.5779<br>(0.6278)
bone	0.0978<br>(0.1332)	0.2000<br>(0.2500)	0.3028<br>(0.3994)	0.3991<br>(0.4812)	0.4808<br>(0.6082)
brain	0.1771<br>(0.2143)	0.2608<br>(0.3393)	0.3852<br>(0.4391)	0.5000<br>(0.5637)	0.6214<br>(0.6667)
breast	0.1031<br>(0.1601)	0.2000<br>(0.2774)	0.3737<br>(0.4245)	0.5000<br>(0.5444)	0.6000<br>(0.6594)
liver	0.1364<br>(0.1556)	0.2479<br>(0.3000)	0.3667<br>(0.4091)	0.4651<br>(0.5172)	0.5783<br>(0.6071)
ovary	0.0972<br>(0.1029)	0.2000<br>(0.2032)	0.3189<br>(0.3333)	0.4389<br>(0.4689)	0.5437<br>(0.5731)
overall	0.1287<br>(0.1443)	0.2549<br>(0.2727)	0.3500<br>(0.3888)	0.4510<br>(0.4949)	0.5573<br>(0.5908)
pancreatic	0.0616<br>(0.0900)	0.1462<br>(0.1911)	0.2581<br>(0.2968)	0.3969<br>(0.4345)	0.5282<br>(0.5550)
prostate	0.1064<br>(0.1161)	0.2174<br>(0.2351)	0.3333<br>(0.3494)	0.4474<br>(0.4639)	0.5506<br>(0.5790)
skin	0.0588<br>(0.0981)	0.1926<br>(0.2063)	0.2992<br>(0.3389)	0.4180<br>(0.4478)	0.5521<br>(0.6000)
uterus	0.1174<br>(0.1429)	0.2000<br>(0.2356)	0.3333<br>(0.3604)	0.4453<br>(0.5000)	0.5319<br>(0.5714)

Table S4. The stem-loop based models: the metrics for the best stem-loop-based models for each cancer type.

	Best stem-loop-based model					
Cancer type	Aggregation level	Labeling	Probability quantile	Median recall	Lift of recall	Median test AUC
skin	1mb	0.5	0.10	0.2	2.00	0.55
overall	500kb	0.1	0.05	0.33	6.67	0.72
prostate	20kb	0.1	0.05	0.10	2.05	0.55
uterus	1mb	0.5	0.05	0.20	4.00	0.65
bone	100kb	0.05	0.1	0.40	4.00	0.68
brain	20kb	0.1	0.05	0.17	3.33	0.63
breast	10kb	0.01	0.10	0.33	3.33	0.63
ovary	1mb	1	0.05	0.09	1.82	0.59
pancreatic	1mb	0.5	0.1	0.17	1.67	0.58
blood	50kb	0.1	0.05	0.18	3.64	0.66
liver	50kb	0.05	0.05	0.29	5.72	0.71

Table S5. The quadruplex-based models: the median and the third quantile of recall (in brackets) among all datasets for each cancer type and quantile.

Cancer type	0.1	0.2	0.3	0.4	0.5
blood	0.1098<br>(0.1380)	0.2123<br>(0.2500)	0.3274<br>(0.3601)	0.4171<br>(0.4710)	0.5146<br>(0.5647)
bone	0.2470<br>(0.3031)	0.4821<br>(0.5667)	0.6515<br>(0.7000)	0.7434<br>(0.8000)	0.7639<br>(0.8000)
brain	0.1381<br>(0.1660)	0.2775<br>(0.3058)	0.3743<br>(0.4000)	0.4701<br>(0.5384)	0.5408<br>(0.6287)
breast	0.3333<br>(0.4000)	0.4545<br>(0.6000)	0.5556<br>(0.6000)	0.6000<br>(0.6833)	0.6336<br>(0.7113)
liver	0.1429<br>(0.2353)	0.2500<br>(0.3000)	0.3451<br>(0.3750)	0.4235<br>(0.4643)	0.5097<br>(0.5412)
ovary	0.1181<br>(0.1616)	0.2183<br>(0.2980)	0.3344<br>(0.3976)	0.4223<br>(0.4860)	0.5180<br>(0.5474)
overall	0.1373<br>(0.1909)	0.2549<br>(0.2864)	0.3400<br>(0.4000)	0.4510<br>(0.5598)	0.5296<br>(0.6000)
pancreatic	0.0481<br>(0.1179)	0.1903<br>(0.2222)	0.2878<br>(0.3292)	0.4014<br>(0.4434)	0.5139<br>(0.5493)
prostate	0.0000<br>(0.0697)	0.1573<br>(0.1836)	0.2778<br>(0.3060)	0.3824<br>(0.4000)	0.4928<br>(0.5000)
skin	0.1429<br>(0.1990)	0.2609<br>(0.3000)	0.3739<br>(0.4000)	0.4851<br>(0.5147)	0.5684<br>(0.6164)
uterus	0.1489<br>(0.2000)	0.3077<br>(0.3957)	0.4000<br>(0.4286)	0.4659<br>(0.5000)	0.5532<br>(0.5714)

Table S6. The quadruplex-based models: the metrics for the best quadruplex-based models for each cancer type.

	Best quadruplex-based model					
Cancer type	Aggregation level	Labeling	Probability quantile	Median recall	Lift of recall	Median test AUC
skin	1mb	0.5	0.05	0.2	4	0.64
overall	20kb	0.01	0.15	0.5	3.3	0.65
prostate	50kb	0.05	0.15	0.27	1.8	0.56
uterus	100kb	0.05	0.05	0.2	4	0.61
bone	20kb	0.01	0.05	0.5	10	0.86
brain	20kb	0.1	0.05	0.17	3.3	0.56
breast	500kb	0.1	0.10	1	10	0.94
ovary	500kb	0.1	0.05	0.33	6.67	0.68
pancreatic	100kb	0.05	0.35	0.6	1.71	0.57
blood	20kb	0.01	0.05	0.2	4	0.65
liver	10kb	0.01	0.05	0.22	4.4	0.55

Table S7. The joint stem-loop and quadruplex-based models: the median and the third quantile of recall (in brackets) for each cancer type and quantile.

Cancer type	0.1	0.2	0.3	0.4	0.5
blood	0.1364<br>(0.1619)	0.2542<br>(0.3125)	0.3687<br>(0.4023)	0.4852<br>(0.5000)	0.5888<br>(0.6328)
bone	0.2420<br>(0.3031)	0.4365<br>(0.5000)	0.5179<br>(0.6000)	0.6369<br>(0.6974)	0.7208<br>(0.7833)
brain	0.1689<br>(0.2143)	0.2793<br>(0.3333)	0.3750<br>(0.4286)	0.5000<br>(0.5354)	0.6250<br>(0.6589)
breast	0.3125<br>(0.3660)	0.4490<br>(0.5000)	0.5714<br>(0.6182)	0.6250<br>(0.7000)	0.7000<br>(0.7483)
liver	0.1364<br>(0.1724)	0.2530<br>(0.2759)	0.3556<br>(0.4000)	0.4699<br>(0.5000)	0.5652<br>(0.6000)
ovary	0.0988<br>(0.1111)	0.2061<br>(0.2440)	0.3169<br>(0.3755)	0.4124<br>(0.4437)	0.5189<br>(0.5425)
overall	0.1324<br>(0.1618)	0.2451<br>(0.2759)	0.3557<br>(0.3827)	0.4563<br>(0.4826)	0.5714<br>(0.6000)
pancreatic	0.0597<br>(0.0952)	0.1416<br>(0.1852)	0.2620<br>(0.3074)	0.3932<br>(0.4136)	0.5240<br>(0.5524)
prostate	0.0960<br>(0.1141)	0.2174<br>(0.2366)	0.3421<br>(0.3567)	0.4498<br>(0.4663)	0.5583<br>(0.5748)
skin	0.1000<br>(0.1512)	0.2000<br>(0.2424)	0.3438<br>(0.3842)	0.4500<br>(0.4685)	0.5492<br>(0.5753)
uterus	0.1591<br>(0.2000)	0.2857<br>(0.3267)	0.3864<br>(0.4167)	0.4615<br>(0.5000)	0.5673<br>(0.5907)

Table S8. The joint stem-loop and quadruplex-based models: the metrics for the best joint stem-loop and quadruplex-based models for each cancer type.

	Best stem-loop and quadruplex-based model					
Cancer type	Aggregation level	Labeling	Lift of recall	Probability quantile	Median recall	Median test AUC
skin	100kb	0.5	2.16	0.1	0.22	0.56
overall	1mb	0.5	3.33	0.05	0.17	0.68
prostate	100 kb	0.5	1.67	0.05	0.08	0.54
uterus	1mb	0.5	4	0.05	0.2	0.64
bone	20kb	0.01	10	0.05	0.5	0.83
brain	100kb	0.05	5	0.05	0.25	0.67
breast	500kb	0.1	10	0.1	1	0.93
ovary	500kb	0.1	2.22	0.15	0.33	0.65
pancreatic	1mb	0.5	1.33	0.15	0.2	0.58
blood	50kb	0.1	4	0.05	0.2	0.67
liver	50kb	0.05	5.72	0.05	0.29	0.73

Table S9. Used fields in cancer breakpoints data table

Field name	ield description
donor_id	Identification key of a donor
sample_id	Identification key of specimen which was obtained from a donor. It is possible that data contain several specimens from one donor
sv_id	Identification key of a particular structural variant observation
chr_from	Label of the donor chromosome which contains variation
chr_from_bkpt	Position (in base pairs) of breakpoint of the variation on the donor chromosome
chr_from_range	Number of bases around chr_from_bkpt that may contain the real breakpoint
chr_to	Label of the acceptor chromosome which contains variation
chr_to_bkpt	Position (in base pairs) of breakpoint of the variation on the acceptor chromosome
chr_to_range	Number of bases around chr_to_bkpt that may contain the real breakpoint

Fields “chr_from_bkpt” and “chr_from_range” describe position of breakpoint in chromosome (“chr_from” / “chr_to”). Inaccuracy of the position is shown in “chr_from_bkpt” / “chr_to_range” fields.

Table S10. The number of new cancer cases by type.

Cancer type	Number of new cases in 2012	Ratio
brain	256	0,03971455166
blood	918	0,14241390009
bone	44	0,00682593857
uterus	848	0,13155445237
liver	782	0,12131554452
prostate	1112	0,17251008377
skin	232	0,03599131244
ovary	239	0,03707725721
pancreatic	338	0,05243561899
breast	1677	0,26016134037
